# Catalysis-Induced Highly-Stable Interface on Porous Silicon for High-Rate Lithium-Ion Batteries

**DOI:** 10.1007/s40820-025-01701-8

**Published:** 2025-03-26

**Authors:** Zhuobin Han, Phornphimon Maitarad, Nuttapon Yodsin, Baogang Zhao, Haoyu Ma, Kexin Liu, Yongfeng Hu, Siriporn Jungsuttiwong, Yumei Wang, Li Lu, Liyi Shi, Shuai Yuan, Yongyao Xia, Yingying Lv

**Affiliations:** 1 Research Centre of Nanoscience and Nanotechnology, Shanghai University, Shanghai, 200444 People’s Republic of China; 2 Department of Chemistry, Faculty of Science, Silpakorn University, Nakhon Pathom, 73000 Thailand; 3 Sinopec Shanghai Research Institute of Petrochemical Technology Co., Ltd., Shanghai, 201208 People’s Republic of China; 4 Department of Chemistry and Center of Excellence for Innovation in Chemistry Faculty of Science, Ubon Ratchathani University, Ubon Ratchathani, 34190 Thailand; 5 National University of Singapore (Chongqing) Research Institute, Chongqing, 401123 People’s Republic of China; 6 Department of Chemistry, Fudan University, Shanghai, 200433 People’s Republic of China; 7 Emerging Industries Institute Shanghai University, Jiaxing, 314006 Zhejiang People’s Republic of China; 8 Program in Bioinformatics and Computational Biology, Graduate School, Chulalongkorn University, Bangkok, 10330 Thailand

**Keywords:** Catalytic interface, Mesoporous, Inorganic-rich SEI, Silicon anode, Lithium-ion batteries

## Abstract

Silicon stands as a key anode material in lithium-ion battery ascribing to its high energy density. Nevertheless, the poor rate performance and limited cycling life remain unresolved through conventional approaches that involve carbon composites or nanostructures, primarily due to the un-controllable effects arising from the substantial formation of a solid electrolyte interphase (SEI) during the cycling. Here, an ultra-thin and homogeneous Ti doping alumina oxide catalytic interface is meticulously applied on the porous Si through a synergistic etching and hydrolysis process. This defect-rich oxide interface promotes a selective adsorption of fluoroethylene carbonate, leading to a catalytic reaction that can be aptly described as “molecular concentration-in situ conversion”. The resultant inorganic-rich SEI layer is electrochemical stable and favors ion-transport, particularly at high-rate cycling and high temperature. The robustly shielded porous Si, with a large surface area, achieves a high initial Coulombic efficiency of 84.7% and delivers exceptional high-rate performance at 25 A g^−1^ (692 mAh g^−1^) and a high Coulombic efficiency of 99.7% over 1000 cycles. The robust SEI constructed through a precious catalytic layer promises significant advantages for the fast development of silicon-based anode in fast-charging batteries.

**Supplementary Information:**

The online version contains supplementary material available at 10.1007/s40820-025-01701-8.

## Highlights

- In situ etching and co-growth of ultra thin defect-rich oxide layers on porous silicon.
- Through the construction of nano-sized catalytic interface, the electrolyte addition FEC would decompose to a LiF-rich solid electrolyte interphase (SEI).
- The robust SEI on porous structured silicon performs high-rate stability at varied temperatures.

**Supplementary Information:**

The online version contains supplementary material available at 10.1007/s40820-025-01701-8.

## Graphical abstract

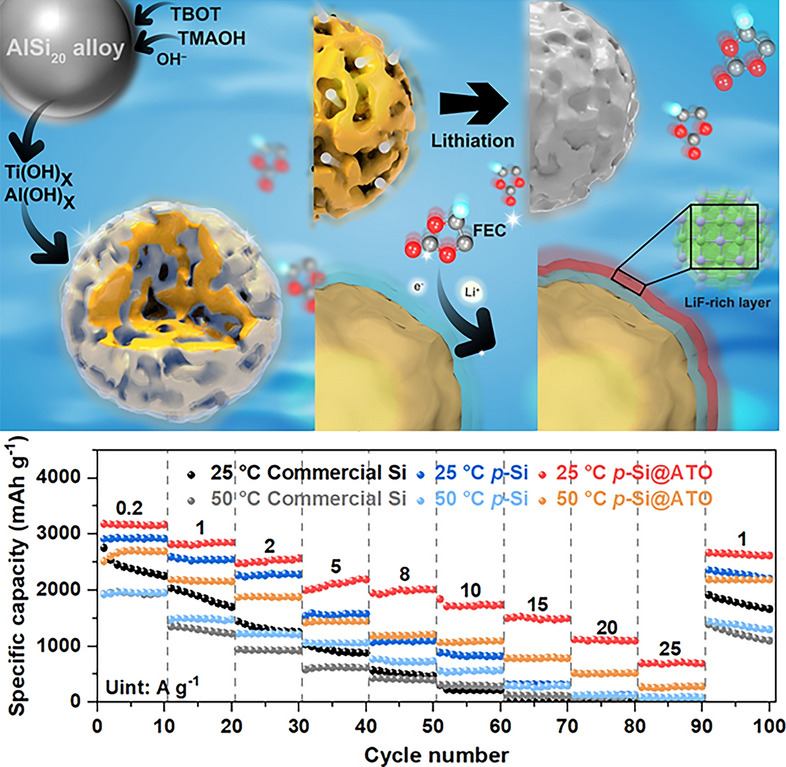

## Introduction

Silicon anodes, with high theoretical specific capacity of 3579 mAh g^−1^ hold a predominate position in the commercial market for high energy density lithium-ion batteries. However, huge volume change of Si (~ 300%) during lithiation/delithiation, always leads to capacity degradation [1, 2], demonstrating low power density and limited cycling life, especially at high temperature. On the interface of silicon, the solid electrolyte interphase (SEI) is fragile and unstable, which could not sustain the huge volume change of the silicon, would be continuous destroyed and re-formation at the repeating cycles [3, 4].

Nano-structured Si is developed to improve the ion transportation at high current density, for example, nanoparticle, nanowire, porous structure or composites. Although structural stability is improved, severe chemical reactions at the interface cannot be fully addressed through the above strategy. The large specific surface area of nanostructures and repeating side reaction would consume the electrolyte, lead to fast capacity degradation [1, 5]. The adverse reactions would accelerate at high temperatures, might precipitate thermal runaway, posing significant safety risks [6, 7]. In essence, designing the stable SEI with specific composition and structure could be achieved through electrolyte modulation and electrode design. However, devising an efficacious interfacial protection layer between the electrode and electrolyte is a critical challenge.

Recently, the interaction between electrodes and electrolytes have gathered increasing attention [8–11]. The deployment of high-concentration or localized high-concentration electrolytes can modulate the solvation structure of lithium ions, thereby engendering a stable SEI [12, 13]. Meanwhile, the incorporation of electrolyte additives to improve the interfacial stability of the electrode, such as vinylene carbonate (VC) and fluoroethylene carbonate (FEC) have been shown [14–16]. FEC, in particular, is widely used due to its higher reduction potential compared with other electrolyte components [17–19], which is decomposed to form a stable LiF-rich SEI. LiF is considered as a key inorganic component of SEI, owing to its high chemical stability and electronic insulating [20]. Most of the research is focused on the formation of an SEI with specific components through electrolyte modulation, while the establishment of a robust SEI layer on anode materials through a surface engineering strategy is rarely studied.

The redox of the electrolyte have diverse pathways and lead to varied reactivity during the presence of varied interface. Meanwhile, transition metals with special d-orbitals are conjectured to catalyze the electrolyte's decomposition [21, 22]. Furthermore, the introduction of surface defects can effectively improve the electrochemical activity by modulating functional groups or incorporating heteroatoms [23–25]. It is widely acknowledged that adsorption is prior to catalysis. Thus, through the deliberate design of electrode structures and electrolyte compositions, targeted solvent molecules can be specifically adsorbed onto the inner Helmholtz plane (IHP) of the electric double layer at the electrode surface. Subsequently, solvent molecules in IHP are preferentially decomposed to form the SEI with specific composition [26, 27]. Thus, a catalytic process achieved through precise synthetic control of the electrode is urgently required, and a robust SEI can be realized.

Therefore, how to solve these problems simultaneously is a major challenge. Through a spontaneous in situ etching and co-growth process, an amorphous alumina-titanium oxide layer is coated on the surface of as-formed porous Si. As prospect, a preferential adsorption of FEC, together with the catalytic effect from defect-rich oxide layer, would generate an inorganic LiF-rich SEI through a fasten-catalytic process. More importantly, from room temperature to high temperature, the LiF-rich interface perform excellent cycling stability (capacity retention is 79.8% after 1000 cycles at 5 A g^−1^), high-rate performance (the capacity reaches 692 mAh g^−1^ even at 25 A g^−1^) and high temperature stability (capacity retention is 80.0% after 500 cycles at 50 °C). This comprehensive understanding of the interface dynamics and catalytic reaction-induced robust SEI processes is vital for the ongoing development and optimization of anode materials for high-performance lithium-ion batteries at high temperature.

## Experimental Section

### Materials Preparation

#### Synthesis of p-Si@ATO

Typically, 0.2 g of commercial AlSi_20_ alloy (Changsha Tianjiu metal material Co., China) is dispersed in 100 mL of ethanol, then 1.6 g of tetramethylammonium hydroxide (TMAOH, dissolved in 1 mL of water) that provides an alkaline environment and 15 mg of tetrabutyl orthotitanate (TBOT) were added sequentially to the above solution. After stirring at 60 °C for 12 h, 1.6 g of TMAOH was added and stirred for another 12 h. The resulting product was centrifuged with ethanol for three times and dried overnight at 80 °C under vacuum. The obtained powder was dispersed into 0.1 M HCl etched 24 h to remove residual Al. Then washed with deionized water to neutral, finally, centrifuged with ethanol and dried at 80 °C under vacuum.

#### Synthesis of p-Si

1.0 g of commercial AlSi_20_ alloy was dispersed into 0.1 M HCl etched 24 h to remove Al. Then, washed with water to neutral, finally, centrifuged with ethanol three times and dried overnight at 80 °C under vacuum. (Because Si is not tolerant of strong alkaline environments and it is difficult to fully etch metal Al while also producing a thick layer of alumina, we have opted for acidic etching conditions instead.)

### Material Characterizations

The morphology and microstructure were characterized by field-emission scanning electron microscopy (FESEM, JSM-7500, JEOL) and transmission electron microscopy (TEM, JEM-2100Plus, JEOL). The crystal structure and chemical bonds of materials were characterized by X-ray diffraction (XRD, SmartLab) with Cu K_α_ radiation (*λ*= 0.154 nm), X-ray photoelectron spectroscopy (XPS, Kratos AXIS SUPRA^+^, Shimadzu) equipped with Al K_α_ radiation, and the binding energy was calibrated by the C 1*s* peak (284.8 eV) of contamination carbon. Nitrogen sorption/desorption isotherms were tested at 77 K with the Autosorb-IQ2 (Quantachrome Corporation), the specific surface area (SSA) is measured via BET method and the distribution of pore size is determined using density functional theory (DFT) method. Oxygen vacancies and electron paramagnetism are characterized by electron paramagnetic resonance (EPR) with Bruker A300 at 25 °C. Diffuse reflectance infrared Fourier transform spectroscopy (DRIFTS) of pyridine adsorption is carried out on a Nicolet iS50 spectrometer (Thermo Fisher, USA). The *p*-Si@ATO powder was pretreated under He flow (30 mL min^−1^) at 150 °C for 1 h, the system was cooled down to 25 °C, then the background was collected. Pyridine was injected into the system for 30 min followed by He purging (30 mL min^−1^) for 30 min to remove weakly adsorbed pyridine. Finally, Pyridine adsorption infrared (IR) spectrum was collected at 25 °C. The X-ray absorption spectroscopy (XAS) experiments of *p-*Si@ATO powder were conducted at the Ti *K*-edge on BL14W1 at Shanghai Synchrotron Radiation Facility. Data were acquired in fluorescence mode at room temperature.

### Cell Assembly and Electrochemical Measurements

The working electrode slurry was fabricated by mixing 80 wt% active materials, 10 wt% polyacrylic acid (PAA, average M_n_ = 450,000, Sigma-Aldrich), and 10 wt% acetylene black in N-methyl-2-pyrrolidone (99.9%, Sigma-Aldrich). The as-received slurry was coated on Cu foil and then dried at 80 °C overnight in a vacuum oven. Unless otherwise specified, the loading of the active material was approximately 0.7–1.8 mg cm^−2^. The stainless-steel coin cell (CR2032, Kejing Co., Ltd.) was assembled inside an argon-filled glovebox (O_2_ and H_2_O content < 0.1 ppm) with the synthesized material and lithium foil as counter electrode. In all tests, 1.0 M LiPF_6_ dissolved in ethylene carbonate (EC) and diethyl carbonate (DEC) (1:1 volume ratio) with 15% FEC as electrolyte and Celgard 2500 film as separator were used. All cells were rested 24 h before cycle. The cycling and rate performance were evaluated by galvanostatic charge/discharge measurements conducted on the LAND CT3002A (Wuhan LAND electronics Co., Ltd., China) testing system between 0.01 and 2.0 V (versus Li^+^/Li) at 25/50 °C. For high temperature cycling, the cell is activated two cycles at 0.2, 0.5, 1, and 2 A g^−1^, respectively. For high-rate cycling, the cell is activated two cycles at 0.2, 0.5, 1, 2, 5, 8, 10, and 15 A g^−1^, respectively. For pouch cell, the active material loading of anode is 0.5 mg cm^−2^, LiFePO_4_ is 10.2 mg cm^−2^, N/P≈0.85. The voltage range is 2.5–3.9 V, and 1 C = 160 mAh g^−1^. Neither the anode nor cathode electrodes are pre-lithiated.

Electrochemical impedance spectroscopy (EIS) with a frequency range of 1.0 M–0.01 Hz and a voltage amplitude of 5.0 mV were conducted on Gamry Reference 600+ . For in situ EIS, EIS data is collected at different voltages, during the discharge/charge process at 0.2 A g^−1^. The obtained EIS data were analyzed by distribution of relaxation times (DRT) after satisfying the Kramers-Kronig relationship. Nyquist diagrams typically necessitate equivalent circuit models for the analysis of electrochemical processes. This presents a significant challenge in accurately distinguishing individual electrochemical reactions amid multifaceted interface interactions. DRT analysis enables the direct determination of the time constants (*τ*) associated with the primary electrochemical processes, thereby streamlining impedance analysis and notably enhancing the precision of kinetic interpretations concerning time scales. The DRT describes the time relaxation characteristics of the electrochemical system analyzed, isolates the processes with different time constants, and it gives direct access to the distribution of the timescales. During the actual measurement, there is noise interference, in situ EIS and DRT results are only used to show the trends of each process as a semiquantitative method instead of using it as quantitative method. We used the open MATLAB code shared online by Prof. Francesco Ciucci (https://github.com/ciuccislab/DRTtools).

### Theoretical Simulations

All calculations were performed based on the DFT-D3, as implemented in Vienna ab initio simulation package (VASP). The generalized gradient approximation (GGA) with Perdew-Burke-Ernzerhof (PBE) functional was used to describe the electron exchange–correlation. In this case, a supercell of size 3 × 3 × 1 was used to simulate Li adsorption. The vacuum spacing along the z-axis was 15 Å to avoid the interaction between the layer and its periodically repeated images. With a cutoff energy of 450 eV, the structures relaxation has no any symmetry constraints. The convergence threshold for energy was set to 1 × 10^−5^ eV and the force was chosen as 0.02 eV Å^−1^. The k-points were set as gamma 111.

## Results and Discussion

### Structure of Defect-Rich Oxides Layer

**Fig. 1:**
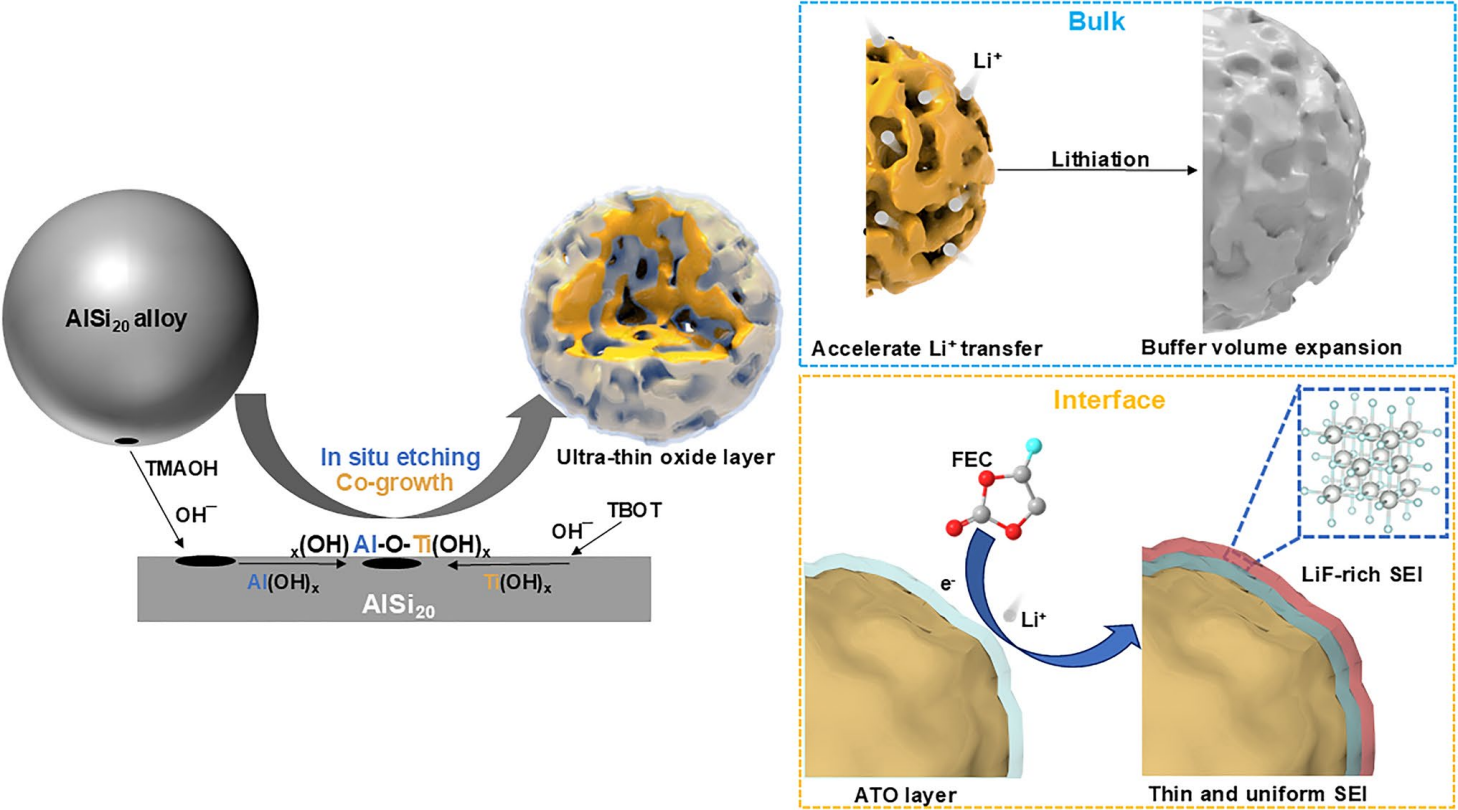
Schematic illustration of the in situ etching and co-growth procedures for *p*-Si@ATO and the corresponding porous structure and surface property

The AlSi_20_ microsphere with a particle size approximately 1.2 μm (Fig. S1) are used as initial material directly. Through a controlled in situ etching and co-growth process in an alkaline environment, the mild etching and simultaneous oxidation of aluminum occur on the surface of the alloy microsphere, in conjunction with hydrolysis of trace amount of TBOT. Thus, porous Si with a homogeneous alumina and titanium oxide layer (referred to as *p*-Si@ATO) can be obtained (Fig. 1). This intricate interplay of etching and hydrolysis coupled with subsequent dehydration culminates in the etching of aluminum and the formation of alumina-titanium oxide layer on the porous Si. Pure porous Si (denoted as *p*-Si) is obtained after the etching of aluminum using the same alloy microspheres.

**Fig. 2:**
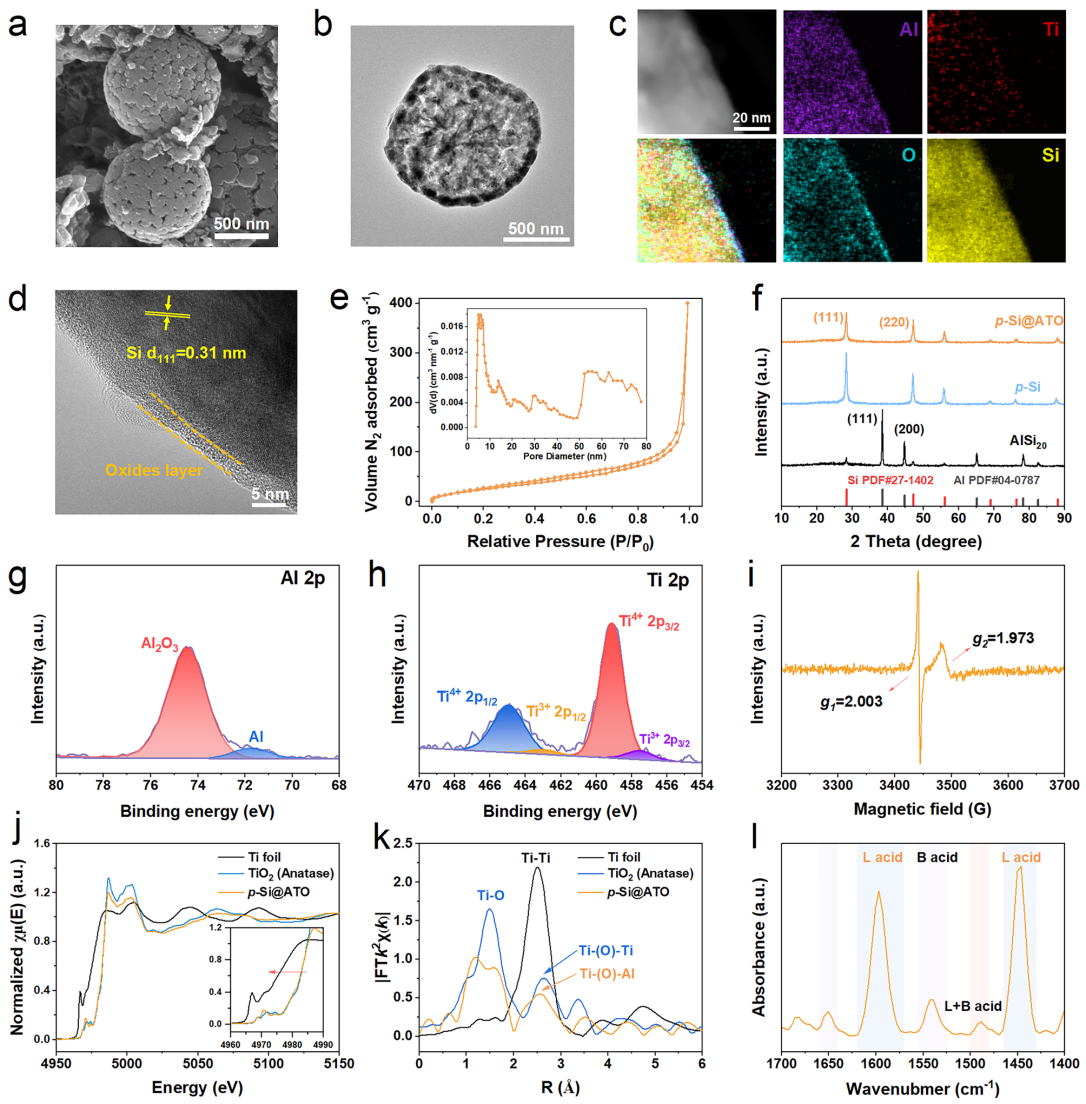
Structural characterizations of *p*-Si@ATO. **a** SEM image of *p*-Si@ATO particle. **b** TEM image of *p*-Si@ATO particle. **c, d** Elemental mapping images and HRTEM image of surface oxides layer. **e** Nitrogen sorption/desorption isotherms and the corresponding pore size distribution of *p*-Si@ATO. **f** XRD pattern of *p*-Si@ATO, *p*-Si and AlSi_20_ alloy. **g, h** High-resolution Al 2*p* and Ti 2*p* XPS of* p*-Si@ATO. **i** EPR spectroscopy of *p*-Si@ATO powder at 25 °C. **j** Normalized Ti *K*-edge XAS spectra of Ti foil, TiO_2_ and *p*-Si@ATO. **k** Fourier transform of EXAFS R-space in *k*^2^-weighted. **l** Pyridine IR spectroscopy of *p*-Si@ATO powder at 25 °C

The as-synthesized *p*-Si@ATO (Fig. 2a) retains the spherical morphology characteristic of the AlSi_20_ alloy, with the surface revealing open pores on the order of tens of nanometers. TEM image (Figs. 2b and S4) displays the unique hollow, dendritic porous architecture of the *p*-Si@ATO. The nitrogen sorption–desorption isotherms (Fig. 2e) exhibit a type IV isotherm [28]. The *p*-Si@ATO reveals a surface area of 112.4 m^2^ g^−1^. The pore size distribution is bimodal, featuring a mesopore diameter centered at about 4.9 nm and a macropore range spanning 50–80 nm. These pores should be raised from the selective etching of aluminum, serving to accommodate the volumetric expansion of Si and enhancing mass transfer rates. The specific surface areas of *p*-Si and commercial Si are 98.5 and 10.1 m^2^ g^−1^, respectively (Figs. S5 and S6). High-resolution TEM (HRTEM) images (Fig. 2c, d) show that the *p*-Si@ATO microsphere is enveloped by a homogeneous amorphous shell approximately 3–5 nm thick which has the Ti, Al and Si elements in the oxide layer on the surface. The presence of distinct lattice fringes with a spacing of 0.31 nm correlates to the (111) crystal plane of Si [29]. The disappearance of the metallic Al peak in the XRD pattern (Fig. 2f) after etching suggests that the metallic Al has been removed from the *p*-Si and *p*-Si@ATO material. Inductively coupled plasma-atomic emission spectrometry analysis quantifies the composition of the *p*-Si@ATO as approximately 85 wt% Si, 5 wt% Al and 0.3 wt% Ti (Table S1).

### Surface Chemical Environment

XPS corroborates the presence of an oxide layer. The natural silicon oxide layer is present on the surfaces of *p*-Si@ATO, *p*-Si and commercial Si (Figs. S9d, f and S10). The Al 2*p* spectra (Fig. 2g) exhibit not only the expected Al_2_O_3_ peak at 74.4 eV but also a minor peak at 71.7 eV, indicating that the metallic Al may contribute partial lithium storage capacity and enhance the internal electronic conductivity. The Ti 2*p* spectrum (Fig. 2h) presents two pronounced peaks at 459.0 and 464.7 eV, corresponding to the 2*p*_3/2_ and 2*p*_1/2_ of Ti^4+^, respectively. Intriguingly, subordinate peaks at 457.2 and 463.1 eV suggest the existence of Ti^3+^ [30]. EPR spectroscopy (Fig. 2i) at 25 °C reveals two distinct paramagnetic signals at *g*_1_ = 2.003 and *g*_2_ = 1.973, ascribed to oxygen vacancies and Ti^3+^, respectively [30, 31]. The presence of Ti^3+^ and oxygen vacancies is acknowledged for their capacity to significantly enhance electronic conductivity and the adsorption of negative charges [31, 32]. Moreover, these oxygen vacancies are instrumental in enhancing the charge transport capabilities of semiconductor electrocatalysts by facilitating the excitation of delocalized electrons to the conduction band, thereby increasing charge carrier density [33].

To decipher the formation of Ti^3+^, the same coating strategy is employed using commercially pure Si instead of the AlSi_20_ alloy for comparative analysis. Unexpectedly, the EPR spectrum (Fig. S11) only identifies oxygen vacancies at *g* = 2.002 with the conspicuous absence of Ti^3+^ signal. Under alkaline conditions, the etching of metallic Al and the hydrolysis of TBOT yield the formation of hydroxides. During the ensuing in situ co-growth phase, these hydroxides are presumed to interlink via Al-O-Ti bonds, forming an ultra-thin amorphous oxide layer. Given the extremely low Ti content (~0.3 wt%), synchrotron XAS in fluorescence mode is performed on the *p*-Si@ATO powder to probe the Al-O-Ti bonds and the precise chemical environment of Ti. The Ti *K*-edge spectrum (Fig. 2j) reveals that the edge energy of *p*-Si@ATO is similar to that of TiO_2_ (anatase), but with a small shift to lower energy and lower peak area, signifying a somewhat reduced valence state of Ti in *p*-Si@ATO straddles in comparison to TiO_2_. This aligns with the Ti^3+^ presence inferred from EPR. The Fourier-transformed extended X-ray absorption fine structure (EXAFS) oscillations (Fig. 2k) delineate the radial distribution of neighboring atoms around the Ti atom, with the peak at approximately 1.5 Å representing the characteristic Ti-O bond [34, 35]. And the intensities of both Ti-O peak and higher order feature (around 2.5 Å) are much reduced compared to those of Ti reference, suggesting a smaller coordination number in *p*-Si@ATO. Meanwhile, *p*-Si@ATO the shift to a smaller R at 2.5 Å, since Al has a smaller radius than Ti, this implies the presence of the Al-O-Ti bond.

The pyridine IR is utilized to investigate the acidic sites on the *p*-Si@ATO surface (Fig. 2l). The distinct absorption bands at 1596.8 and 1446.4 cm^−1^ denote the existence of Lewis (L) acid sites, while the weaker bands at 1542.8 cm^−1^ imply the presence of trace Brønsted (B) acid sites. Additionally, the absorbance near 1488.8 cm^−1^ is indicative of both L and B acid sites on the *p*-Si@ATO surface [36, 37]. Al_2_O_3_, a potent Lewis acid, is extensively applied in catalysis, and it has been proved that the formation of Al-O-Ti bond can enhance its Lewis acidity due to create a greater number of coordinatively unsaturated metal sites [36, 38]. These Lewis acid sites facilitate the adsorption of negative charges, and the establishment of Al-O-Ti bonds on the *p*-Si@ATO surface aids in adsorbing the electrolyte and catalyzing its decomposition. In summation, the surface of porous Si is coated with a 3–5 nm amorphous oxide layer via an in situ etching and co-growth strategy, validating the existence of Al-O-Ti bond. The plentiful Lewis acid sites on the surface favor electrolyte adsorption, while the presence of Ti^3+^ and oxygen vacancies enhance electronic conductivity. These surface characteristics are favorable for the adsorption and mass transfer of the electrolyte in *p*-Si@ATO, thereby engendering a stable SEI. Meanwhile, the abundant mesopore can improve the adsorption of the electrolyte, accelerate the mass transfer rate and buffer the volume expansion.

### Electrochemical Performance

The porous structure offers abundant internal space to accommodate the significant volume changes of Si during lithiation/delithiation. However, the repeated growth of the SEI and the resultant consumption of electrolyte are inevitable problems. To assess the impact of the porous structure and the surface functionality of the ATO layer, detailed electrochemical analyses are conducted on *p*-Si@ATO and commercial Si electrodes. The initial Coulombic efficiency (ICE) of commercial Si is only 79.2% at 25 °C and 70.2% at 50 °C, while the ICE of *p*-Si@ATO at 0.2 A g^−1^ is as high as 84.7% at 25 °C and 73.2% at 50 °C, markedly surpassing the ICE of commercial Si. Notably, pure *p*-Si exhibits lowest ICE, which is only 71.6% at 25 °C and 61.3% at 50 °C, might be due to its extensive specific surface area and lack of effective interface protection (Fig. S14). The improvement in ICE with *p*-Si@ATO is attributed to enhanced interface protection effect of the oxide layer, which reduces side reactions compared to the unstable surface of commercial Si. Furthermore, the initial charge capacity of *p*-Si@ATO (3312 mAh g^−1^ at 25 °C and 2423 mAh g^−1^ at 50 °C) exceeds that of the *p*-Si (2985 mAh g^−1^ at 25 °C and 1987 mAh g^−1^ at 50 °C) and commercial Si (2910 mAh g^−1^ at 25 °C and 1893 mAh g^−1^ at 50 °C). The capacity of the *p*-Si@ATO,* p*-Si and commercial Si at 50 °C is significantly lower than that at room temperature, with rapid capacity degradation at elevated temperatures being attributed to the instability of LiPF_6_-based electrolytes, besides, the interfacial side reactions are more serious at high temperatures, resulting in lower Coulombic efficiency than that at room temperature [39, 40].

**Fig. 3:**
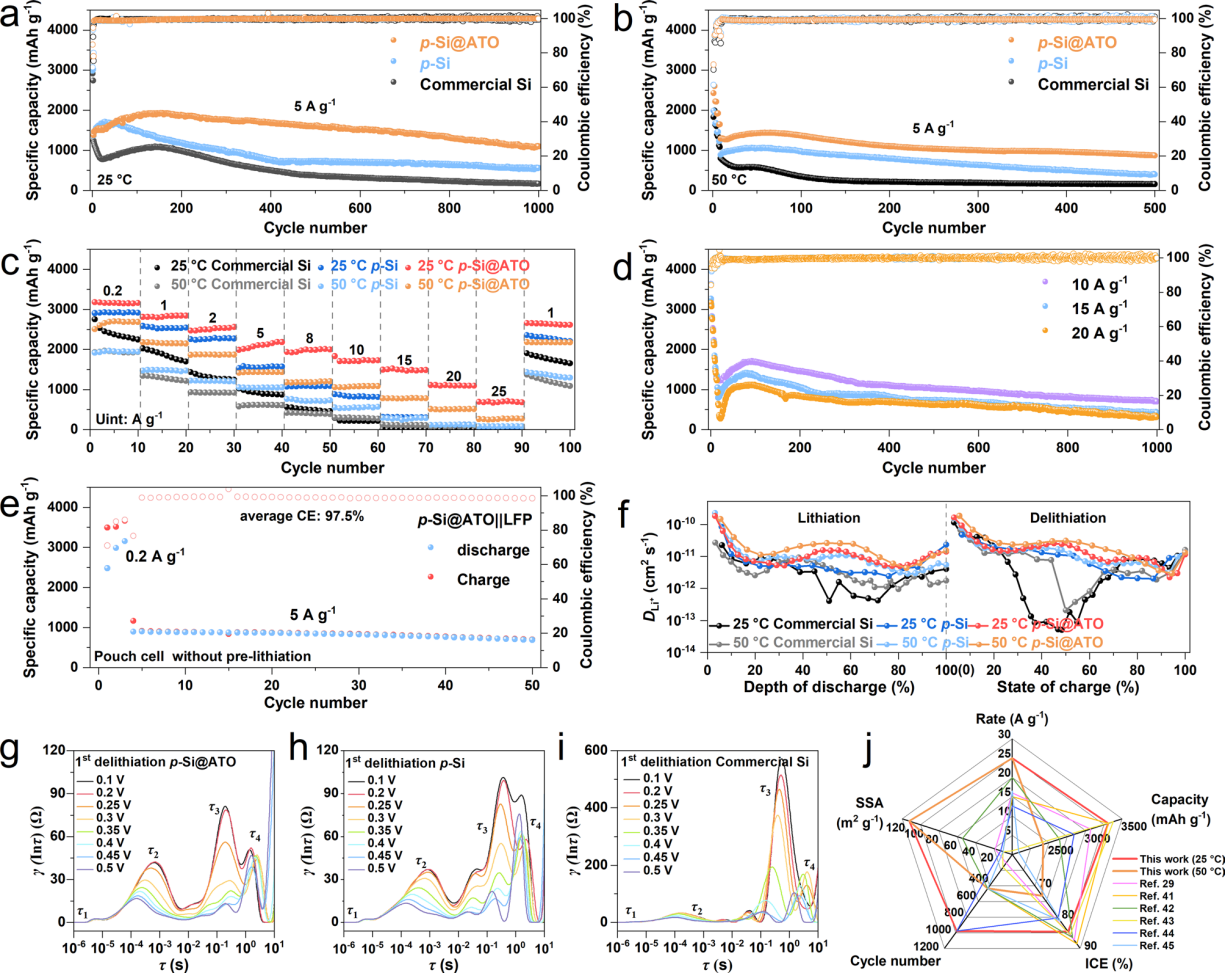
Electrochemical performance of *p*-Si@ATO, *p*-Si and commercial Si anode. **a, b** Long-term cycling performances of *p*-Si@ATO, *p*-Si and commercial Si at 25 and 50 °C. **c** Rate performances of *p*-Si@ATO, *p*-Si and commercial Si. **d** High-rate cycling performances of *p*-Si@ATO anode at 25 °C. **e** Cycling performance of *p*-Si@ATO||LFP pouch cell without pre-lithiation at 25 °C. **f** GITT of *p*-Si@ATO, *p*-Si and commercial Si at 0.2 A g^−1^ after 10 cycles. **g-i** DRT in initial charging process for *p*-Si@ATO, *p*-Si and commercial Si electrode at 25 °C. **j** Comparison of the performances of the *p*-Si@ATO with other Si-based anode materials

At 25 °C (Fig. 3a), after 1000 cycles at 5 A g^−1^, the capacity retention of the commercial Si is only 13.7% (based on the third cycle) and *p*-Si is 41.1%, whereas *p*-Si@ATO demonstrates exceptional capacity retention of 79.8%, which has the average CE of about 99.7% over these 1000 cycles. After 500 cycles at 2 A g^−1^ (Fig. S15), the capacity retention of *p*-Si@ATO is 99.5% which is significantly higher than *p*-Si and commercial Si. At 50 °C (Fig. 3b), the capacity retention of the commercial Si plummets to 42.9% after 100 cycles and *p*-Si to 44.9% after 500 cycles at 5 A g^−1^. In stark contrast, *p*-Si@ATO maintains 67.5% capacity after 500 cycles at 5 A g^−1^. Meanwhile, *p*-Si@ATO maintains 80.0% capacity after 500 cycles at 2 A g^−1^, with average CE around 98.9% (Fig. S16). Furthermore, the rate performance of *p*-Si@ATO is also evaluated at varied of current densities from 0.2 to 25 A g^−1^ (Fig. 3c). At high current densities of 10, 15 and 20 A g^−1^, the capacities are 1730, 1492, and 1103 mAh g^−1^ at 25 °C, respectively. At 50 °C, they are 1076, 784, and 512 mAh g^−1^. Remarkably, even at an extremely high current density of 25 A g^−1^, *p*-Si@ATO maintains a considerable reversible capacity (692 mAh g^−1^ at 25 °C and 270 mAh g^−1^ at 50 °C), whereas the capacity of the *p*-Si and commercial Si drops down to near zero at 20 A g^−1^. The porous structure enables rate performance of *p*-Si@ATO and* p*-Si better than commercial Si, besides, *p*-Si@ATOs with interface protection has best rate performance and capacity. The *p*-Si@ATO electrode also demonstrates excellent high-rate cycling stability, maintaining steady cycling performance over 1000 cycles at 10, 15, and 20 A g^−1^ at 25 °C (Fig. 3d). Such a high reversible capacity, high-rate capability, durable cycling stability and thermal stability outperform the* p*-Si and state-of-the-art Si-base materials reported in literature (Fig. 3j and Table S3) [29, 41–45]. Meanwhile, without any additional conductive agent,* p*-Si@ATO electrode with a high loading anode of 1.8 mg cm^−2^ displays an area capacity of approximately 3.2 mAh cm^−2^ at 0.2 A g^−1^ (Fig. S17). Furthermore, *p*-Si@ATO||LFP full-cell are assembled to test *p*-Si@ATO application performance (Figs. 3e and S18), the cell without any pre-lithiation have a capacity retention of 77.4% after 50 cycles, while the average CE over 50 cycles is 97.5%.

To further elucidate the kinetics of Li^+^ transport in the electrodes, the Li^+^ diffusion coefficient is measured using the galvanostatic intermittent titration technique (GITT). As expected, the Li^+^ diffusion rate is faster at the elevated temperature of 50 °C compared to the standard condition of 25 °C (Fig. 3f). Notably, the *p*-Si@ATO shows a faster diffusion of Li^+^ compared to that of the *p*-Si and commercial Si. Specifically, during the delithiation, the diffusion coefficient of *p*-Si@ATO is about 10^−11^ cm^2^ s^−1^, two orders of magnitude higher than that of commercial Si (10^−13^ cm^2^ s^−1^). This significant difference indicates that the commercial Si suffers from slow delithiation kinetics, which adversely affects its rate performance. In contrast, the mesoporous structure and the presence of active sites on the surface of *p*-Si@ATO are conducive to the rapid adsorption and mass transfer of the electrolyte. These characteristics are crucial in contributing to the material's outstanding high-rate performance and enhanced reversible capacity. EIS (Fig. S21) measurements further confirm the differences in the interfacial properties after the first cycle at a current density of 0.2 A g^−1^. The impedance of *p*-Si@ATO at 25 °C is significantly lower than that of *p*-Si and commercial Si. Meanwhile, the difference in impedance between *p*-Si@ATO and commercial Si diminishes at 50 °C. This suggests that the kinetic processes of *p*-Si@ATO are superior to those of *p*-Si and commercial Si, and that there are variations in the interfacial reactions that occur at different temperatures. More specifically, DRT (Fig. 3g–i) analysis is utilized to investigate the interface evolution at initial charging process. The emergence of peaks at *τ*_1_ below 10^−5^ s which can be ascribed to the contact impedance between the electrode material and the current collector [46]. The *τ*_2_ peak is indicative of the SEI impedance on the lithium (Fig. S22). The *τ*_3_ and *τ*_4_ peaks are representative of the SEI impedance (*R*_SEI_) and charge transfer impedance (*R*_ct_) on Si electrode [47, 48]. The *R*_ct_ and *R*_SEI_ impedances of *p*-Si@ATO are substantially reduced compared to those of *p*-Si and commercial Si, particularly the disparity in *R*_SEI_ on Si electrode side associated with the *τ*_3_ peak. During the delithiation phase between 0.1 and 0.5 V, the *τ*_3_ and *τ*_4_ peaks exhibit a significant lower. This suggests that the SEI constructed on the surface of *p*-Si@ATO has faster delithiation kinetics, which is advantageous for attaining high capacities and high-rate capabilities.

### Interface Analysis on Electrodes

**Fig. 4:**
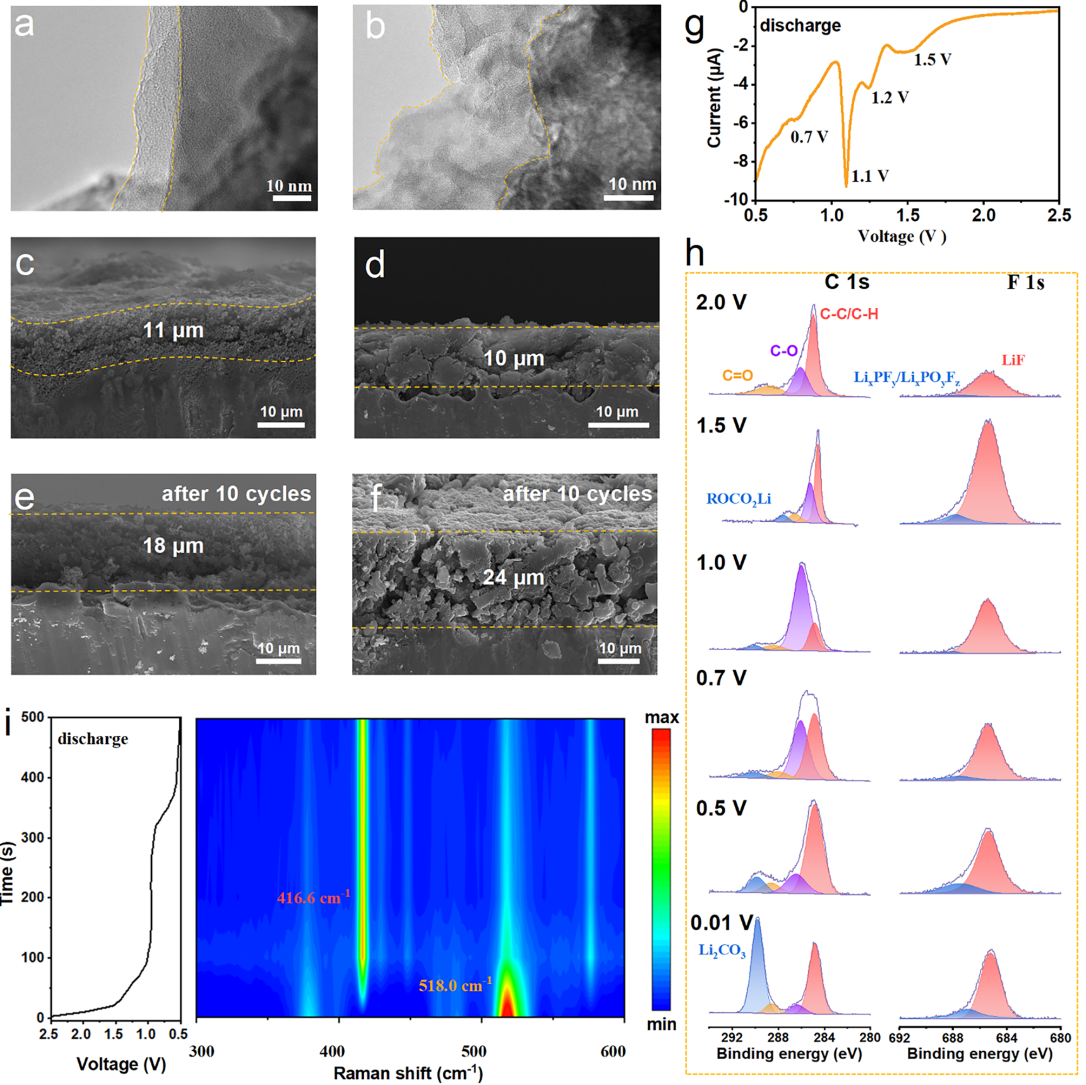
Interface and electrode analysis of *p*-Si@ATO anode. **a**, **b** HRTEM images of *p*-Si@ATO **a** and commercial Si **b** electrodes after 100 cycles. **c-f** Cross-sectional SEM images of *p*-Si@ATO **c**, **d** and commercial Si **e, f** electrodes. **g** LSV in initial discharge process of *p*-Si@ATO at 0.05 mV s^−1^. **h** Quasi in situ XPS of *p*-Si@ATO electrode at different voltages. **i** In situ Raman spectroscopy contour of *p*-Si@ATO electrode in first discharge process at 0.2 A g^−1^

A series of analytical techniques are utilized to provide a detailed comparison of the SEI on *p*-Si@ATO and commercial Si electrodes after cycling. HRTEM images (Fig. 4a, b) reveal that after 100 cycles, the SEI on the *p*-Si@ATO is more uniform and thinner than that on commercial Si, which is beneficial for battery performance as it can provide a stable interface for Li^+^ transport while minimizing resistance and protecting the electrode material. Furthermore, the integrity of the electrodes is investigated. Both types of Si anodes start with a similar initial thickness of about 10 µm (Fig. 4c, d). After 10 cycles, the *p*-Si@ATO electrode thickness increases by about 63.6%, whereas the commercial Si electrode expands by about 140.0% (Fig. 4e, f). This suggests that the porous structure of *p*-Si@ATO mitigates the volume change of Si during lithiation and delithiation processes, which helps maintain the electrode's integrity. On the other hand, the results also indicate that the *p*-Si@ATO electrode maintains a more effective SEI and suffers less mechanical degradation compared to commercial Si, which leads to better electrochemical performance and could potentially translate into longer battery life and higher stability.

Linear sweep voltammetry (LSV) shows multiple reduction peaks between 2.0 and 0.5 V during the initial discharge process at 25 °C (Fig. 4g), which are associated with the reduction and decomposition of the electrolyte that leads to SEI formation [18]. Quasi in situ XPS (Fig. 4h) further confirms the SEI formation process on the *p*-Si@ATO electrode at various discharge voltages. In F 1*s* spectra, a small LiF peak about 685 eV is observed at 2.0 V [49], which grows as the voltage is reduced to 1.5 V. In C 1*s* spectra, alongside peaks corresponding to C-O and C=O increases in intensity, which are indicative of organic SEI components [49]. A small amount of ROCO_2_Li is also detected. A significant increase in the C-O peak at 1.0 V corresponds to the decomposition of EC [50], which is consistent with the LSV. As the discharge continues, the electrolyte decomposition progresses, leading to more side reactions and substantial SEI formation. The LiF peak weakens, while peaks for side reaction products Li_x_PF_y_ and Li_x_PO_y_F_z_ become more prominent (Fig. S24). At 0.01 V, there is a significant increase in Li_2_CO_3_ content. LiF is typically formed at higher voltages (> 1.5 V) than that of Li_2_CO_3_ and organic SEI components, and its presence is crucial for a dense and stable inorganic SEI [4]. In situ Raman spectroscopy (Figs. 4i and S25) supports these findings, showing a peak that may correspond to LiF (416.6 cm^−1^) at approximately 1.8 V [51, 52]. Additionally, the decrease in the intensity of the Si peak (518.0 cm^−1^) at around 1.0 V suggests the formation of the SEI, which diminishes the Si signal [52, 53].

**Fig. 5:**
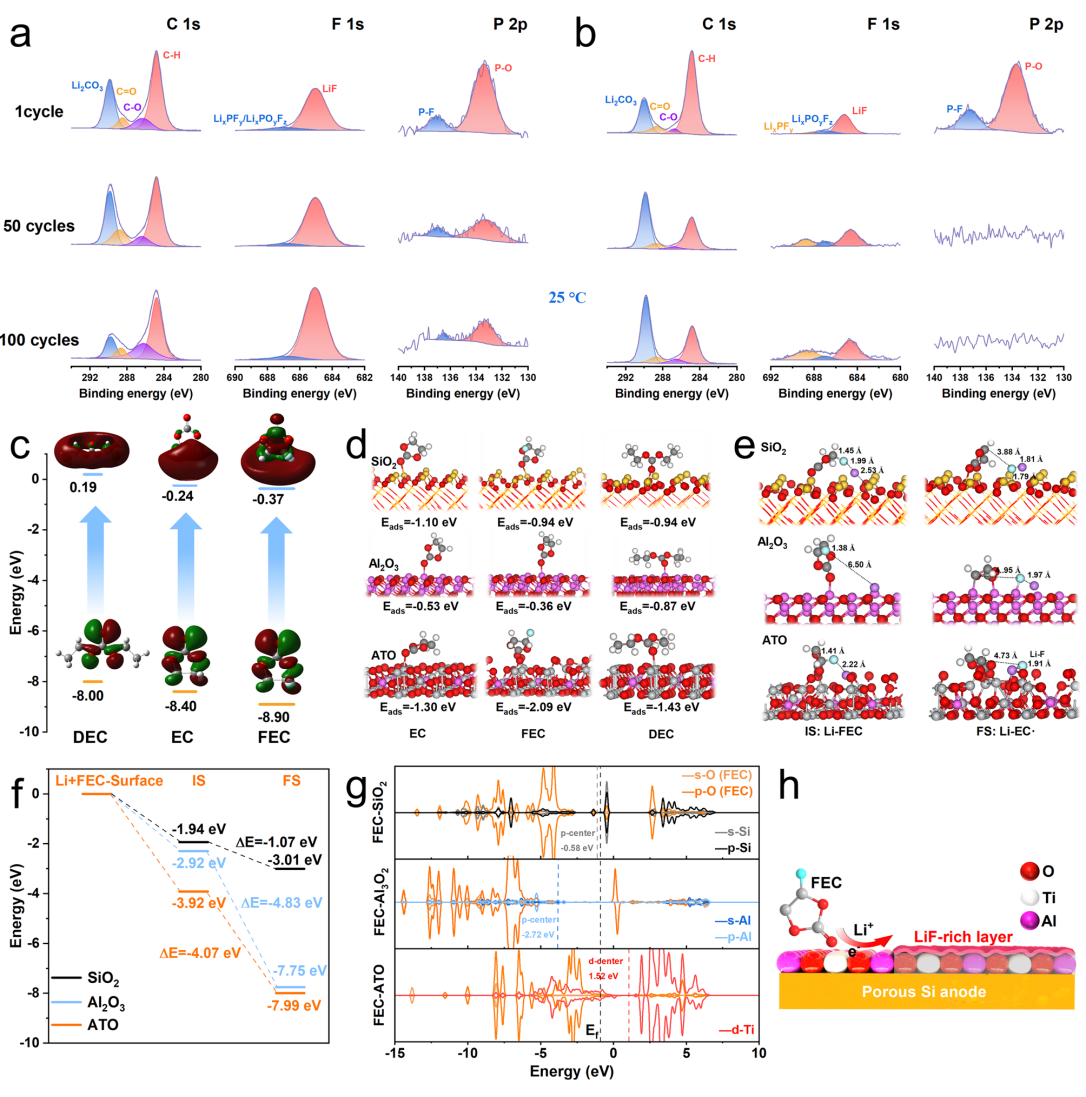
**a, b** C 1*s*, F 1*s* and P 2*p* spectra of *p*-Si@ATO (**a**) and commercial Si (**b**) at 25 °C. All spectra were measured using disassembled half-cell electrodes at 100% SOC. **c** Electronic transition and related *E*_gap_ of solvent molecules. **d** Adsorption configurations of solvent molecules on the different surfaces. **e, f** LiF forming structure and the relative energy profile over the SiO_2_, Al_2_O_3_ and ATO surfaces. **g** PDOS with FEC on SiO_2_, Al_2_O_3_ and ATO surfaces. **h** Schematic illustration of interfacial catalysis about the formation of LiF with FEC solvent over ATO

The SEI forms on the electrode surface during the initial charge–discharge cycles and acts as a barrier that regulates ion transport while preventing further electrolyte decomposition, which are extra unstable on the surfaces of Si. The double-layer structure of the SEI is identified as consists of an inorganic inner layer that includes species such as LiF, Li_2_CO_3_, Li_2_O and an organic outer layer composed of compounds like lithium alkyl carbonates (RCO_2_Li, ROCO_2_Li) [4]. The evolution of the SEI over the course of cycling is investigated by performing XPS on electrodes after 1, 50, and 100 cycles. The spectrums of *p*-Si@ATO and commercial Si electrodes at 25 °C after these various cycle numbers are compared. For *p*-Si@ATO, the F 1*s* spectra (Fig. 5a) reveal an increase in the relative content of LiF with cycling, from 90.6 at% initially, to 92.1 at%, and then to 93.1 at% (Fig. S26). This trend suggests a stable and possibly thickening SEI layer. In contrast, the relative content of LiF on commercial Si electrodes show a decrease from 79.3 to 63.9 at% and further to 54.5 at%, which might indicate a fragile SEI that is breaking down or undergoing continuous reconstruction. This instability can lead to excessive consumption of the electrolyte, gas generation, and the formation of a porous and weak mechanical SEI. LiF is particularly desirable in the SEI due to its electronic insulation properties and mechanical stability, which are beneficial for battery performance and durability. Doping LiF into bulk Si has also been shown to prevent the formation of the crystalline Li_15_Si_4_ phase, which can reduce the capacity loss [54]. It is worth noting higher relative contents of phosphate-containing species (Li_x_PO_y_F_z_ at 687.0 eV and Li_x_PF_y_ at 688.5 eV) on the commercial Si compared to that on the *p*-Si@ATO [49]. These species are often associated with side reactions on the electrode surface, suggesting that commercial Si experiences more severe side reactions that can degrade battery performance over time.

The C 1*s* spectra (Fig. 5a) analysis provides insights into the carbon-containing components of the SEI. For the *p*-Si@ATO electrodes, the relative content of Li_2_CO_3_ at 289.8 eV [49] initially rises and then falls as the number of cycle increases, suggesting formation of Li_2_CO_3_ after a reaction when the electrolyte decomposes, which is part of inorganic layer of SEI, followed by a decomposition of part of Li_2_CO_3_ gradually in the following cycles. The situation with commercial Si is totally different, where, the relative content of Li_2_CO_3_ keeps increasing (Fig. 5b), which could be due to ongoing breakdown and reconstruction of the SEI. The continuous reaction between Li_2_CO_3_ and the electrolyte can produce gases, contributing to a loose SEI [55]. The repeating construction of SEI exacerbate electrolyte decomposition and would lead to limited battery performance and life. The P element relates to the decomposition of the electrolyte component LiPF_6_. In the F 1*s* spectrum, decomposition products such as Li_x_PF_y_ and Li_x_PO_y_F_z_ are detected (Fig. 5b). However, for the commercial Si electrode, P-O bond (133.4 eV) and P-F bond (137.0 eV) signals are weak after the first cycle [56], due probably to a consumption of the electrolyte that leads to a thick organic SEI layer which could obscure the P signals from the XPS measurement. In contrast, the *p*-Si@ATO electrode (Fig. 5a) still shows well-visible P signals even after 100 cycles, indicating a thinner SEI which is a clear indication of less electrolyte consumption and fewer side reactions (Li_2_CO_3_ and organic SEI). In summary, the results suggest that the SEI on the *p*-Si@ATO electrode is better composed with a higher content of LiF and fewer side reaction products. Owing to dense LiF layer and better interface, the SEI on *p*-Si@ATO demonstrates higher stability. The improved SEI structure on *p*-Si@ATO provides enhanced protection against side reactions, contributing to its superior cycling stability and capacity retention compared to commercial Si.

To further elucidate the compositional characteristics and distinctions of the SEI formed on the three silicon-based electrodes, time-of-flight secondary ion mass spectrometry (ToF–SIMS) depth profiling analysis was systematically performed. The corresponding results are presented in Fig. S27. The acquired depth profiles reveal critical insights into the spatial distribution and temporal evolution trends of key ionic species. Specifically, characteristic secondary ions including Si^−^ (representative of Si), C_2_H_2_O^−^ (indicative of organic constituents), LiF_2_^−^ (associated with LiF) and LiCO_3_^−^ (corresponding to Li_2_CO_3_) are monitored as functions of sputtering time. As evidenced by Fig. S27c, the Si⁻ signal in the commercial Si electrode emerges only after 100 s of ion etching, accompanied by a continuous attenuation of organic-related signals (C_2_H_2_O^−^). This observation implies the presence of a relatively thick organic-dominated SEI layer at the surface, with progressively increasing Li_2_CO_3_ content and limited LiF accumulation. In contrast, both *p*-Si and *p*-Si@ATO electrodes exhibit earlier emergence of Si^−^ signals during the etching process. Notably, the *p*-Si@ATO electrode demonstrates substantial LiF enrichment coupled with markedly reduced C_2_H_2_O^−^ content within the SEI. These findings collectively suggest that the *p*-Si@ATO electrode possesses the thinnest SEI layer among the investigated systems, featuring a distinctive architecture comprising a sparse outer organic layer and a dense inorganic-rich interfacial region predominantly composed of LiF, which exhibits an intensified concentration.

### Theoretical Calculation

The interfacial catalytic mechanisms of ATO are verified by DFT calculation. Typically, solvents possessing low LUMO values would undergo prior reduction at the surface to dictate the inner SEI components [57]. FEC manifests the lowest LUMO value, − 0.37 eV, indicative of its preferential decomposition during initial lithiation (Fig. 5c). The three solvent adsorptions are performed over the periodic models of SiO_2_, Al_2_O_3_ and ATO. The adsorption energy of these solvent molecules over the surface of ATO is lower than that of the SiO_2_ and Al_2_O_3_ (Fig. 5d), demonstrating superior adsorption of ATO surface. Notably, the lower adsorption energy of the FEC on ATO (− 2.09 eV) imply that FEC is more easily be accumulated on the surface of ATO. The intermediate optimization of decomposed product LiF's initial state (IS) and final state (FS) with FEC solvent is performed (Fig. 5e). For the IS, the C-F bond in FEC is about 1.45 and 1.41 Å, respectively, on the surface of SiO_2_ and ATO. In comparison, the C-F bond of FEC on the Al_2_O_3_ resulted in the shortest bond length (1.38 Å) due to the far distance of active Al on the surface. Considering FS structural optimizations, after forming Li-F, the C-F bond over three surface models is 3.88–4.95 Å. Longer C-F bonds indicate that it is more likely to break and form LiF [25]. The relative energy diagram of IS and FS on SiO_2_, Al_2_O_3_ and ATO is shown in Fig. 5f. Subsequent to the genesis of Li-F, the energy of the ATO in FS is − 7.99 eV, which is lower than that of the Al_2_O_3_ (− 7.75 eV), and considerably lower that of SiO_2_ (− 3.01 eV). In comparison, the energy profile of LiF with FEC solvent over the ATO surface resulted in more exothermic intermediate energies.

Furthermore, the electronic properties of SiO_2_, Al_2_O_3_, and ATO with the FEC molecule are theoretically investigated using the partial density of state (PDOS, Fig. 5g). In general, shifting the *p*-band or *d*-band centers of active sites is an effective way to regulate electronic and geometric structures because downshifting can aid the desorption of active intermediates [58]. Meanwhile, the upshifting promotes the active intermediates' adsorption, enhancing the active sites' reactivity, which significantly facilitates the activity catalyst's surfaces. The ATO shows the *d*-center at 1.52 eV, crosses the Femi-energy level, which suggests a more active surface in comparison to the SiO_2_ and Al_2_O_3_, where the *p*-center positioned at − 0.58 and − 2.72 eV, respectively. The FEC adsorption on the catalysts resulted in the upshift PDOS in both the *p*-band and *d*-band, as seen in the values of the *p*-center and *d*-center (Fig. S28). The adsorption strength of FEC on ATO is more significant, confirming that FEC is most likely to be decomposed to LiF on the ATO surface, which is an important factor in the perdurable cycling stability of the *p*-Si@ATO electrode (Fig. 5h).

The “molecular concentration-in situ conversion” mechanisms of the functional interface on mesoporous Si microsphere can be described (Fig. 1). Initially, the solvent molecules are adsorbed on the electrode surface, similar with supercapacitor, which are accumulated on the surfaces. Enrich of the FEC is then directly catalyzed to the LiF-rich SEI. Compared with commercial Si, the *p*-Si@ATO proved to with lower adsorption energy with FEC. Moreover, the LiF-rich SEI derived from ultra-thin oxide layer reduces the resistance of mass transfer, which is conducive to improving the high-rate performance. As the synergetic effect of the both molecular concentration and in situ conversion of functional interface, the *p*-Si@ATO is proved to own LiF-rich robust and thinner inorganic SEI layer than other Si-based materials. The high-rate and high temperature stabilities of *p*-Si@ATO are facilitated by the effective resolution of electrolyte depletion at the surface functional interface, while the mesopore structure of the microsphere acts as a buffer, safeguarding against cracking and preserving electrode integrity.

## Conclusion

In this work, an ultra-thin catalytic interface on mesoporous Si has been innovatively developed through an in situ etching and co-growth process. Stemming from the catalytic effect of defect-rich interface, and the concentrated accumulation of FEC within the mesopores, a uniform LiF-rich robust SEI would be formed. The as-protected mesoporous Si microsphere has highly reversible capacity at high current density 25 A g^−1^ (692 mAh g^−1^ at 25 °C and 270 mAh g^−1^ at 50 °C) and high-temperature capacity retention (80.0%) over 500 cycles at 2 A g^−1^. Particularly, this Si anode performs long cycling stability as long as 1000 cycles even at an elevated current density of 20 A g^−1^. Consequently, this strategy points out the effective way to mitigate capacity degradation in Si, particularly at harsh condition, satisfying the critical requirement for a high safety and long durable anode with both high energy density and high power density.

## Supplementary Information

Below is the link to the electronic supplementary material.

Supplementary file1 (DOCX 8223 kb)
