# Supplementary material for: Catalysis-Induced Highly-Stable Interface on Porous Silicon for High-Rate Lithium-Ion Batteries
Source: Nanomicro Lett. 2025 Mar 26;17:200. doi: 10.1007/s40820-025-01701-8 (PMC11937483; doi:10.1007/s40820-025-01701-8)
Supplement: Supplementary file 1 — Supplementary file1 (DOCX 8223 kb) [file 40820_2025_1701_MOESM1_ESM.docx]

Supporting Information for

**Catalysis-Induced Highly-Stable Interface on Porous Silicon for High-Rate Lithium-Ion Batteries**

Zhuobin Han^1,†^, Phornphimon Maitarad^1,8,†^, Nuttapon Yodsin^2^, Baogang Zhao^1^, Haoyu Ma^1^, Kexin Liu^1^, Yongfeng Hu^3^, Siriporn Jungsuttiwong^4^, Yumei Wang^5^, Li Lu^5^, Liyi Shi^1,7^, Shuai Yuan^1^, Yongyao Xia^6,^* and Yingying Lv^1,^*

^1^ Research Centre of Nanoscience and Nanotechnology, Shanghai University, Shanghai 200444, People’s Republic of China

^2^ Department of Chemistry, Faculty of Science, Silpakorn University, Nakhon Pathom 73000, The Kingdom of Thailand

^3^ Sinopec Shanghai Research Institute of Petrochemical Technology Co., Ltd., Shanghai 201208, People’s Republic of China

^4^ Department of Chemistry and Center of Excellence for Innovation in Chemistry Faculty of Science, Ubon Ratchathani University, Ubon Ratchathani 34190, The Kingdom of Thailand

^5^ National University of Singapore (Chongqing) Research Institute, Chongqing 401123, People’s Republic of China

^6^ Department of Chemistry, Fudan University, Shanghai 200433, People’s Republic of China

^7^ Emerging Industries Institute Shanghai University, Jiaxing, Zhejiang 314006, People’s Republic of China

^8^Program in Bioinformatics and Computational Biology, Graduate School, Chulalongkorn University, Bangkok 10330, The Kingdom of Thailand

^†^ Zhuobin Han and Phornphimon Maitarad contributed equally to this work.

*Corresponding authors. E-mail: [yyinglv@shu.edu.cn](mailto:yyinglv@shu.edu.cn) (Yingying Lv); [yyxia@fudan.edu.cn](mailto:yyxia@fudan.edu.cn) (Yongyao Xia)

**Supplementary Figures and Tables**
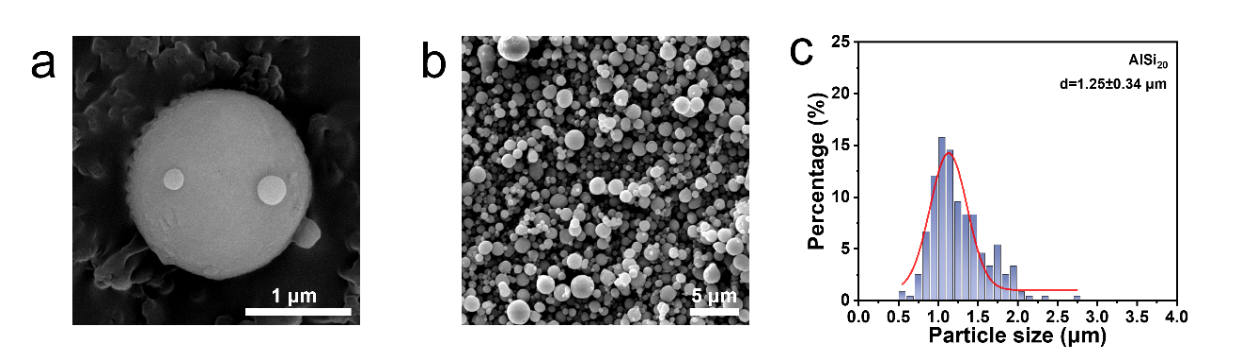


**Fig. S1** (**a, b**) SEM images of pristine **AlSi_20_ alloy**; (**b**) The particle size distribution of pristine **AlSi_20_ alloy**


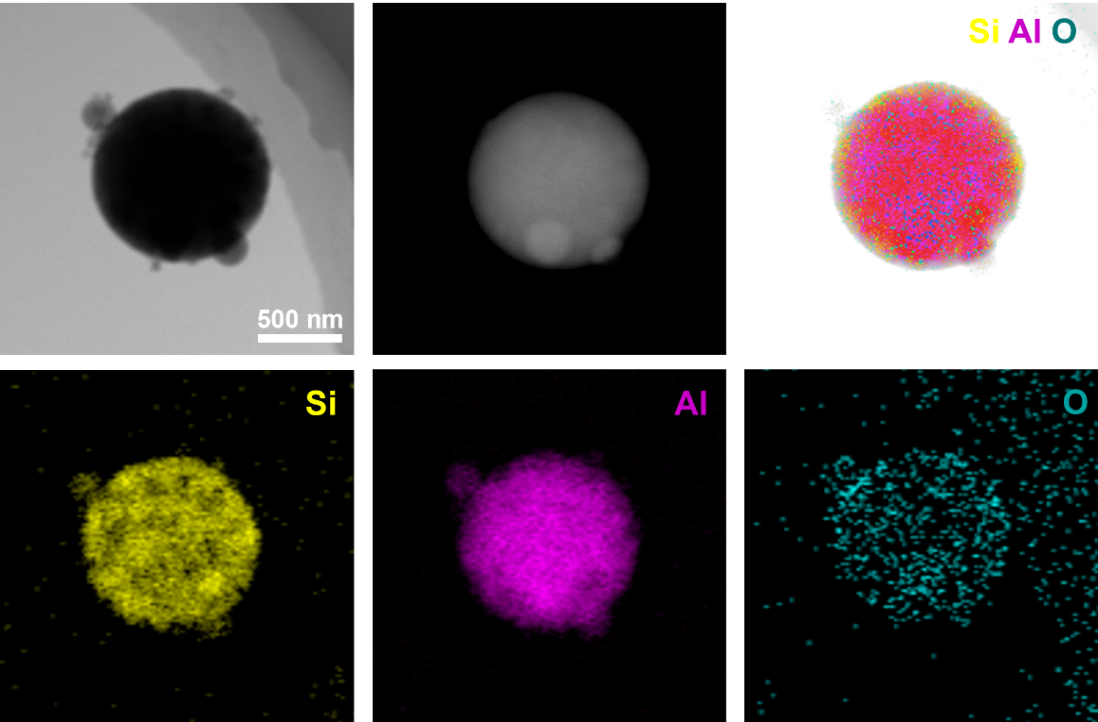


**Fig. S2** TEM and elemental mapping images of **AlSi_20_ alloy**


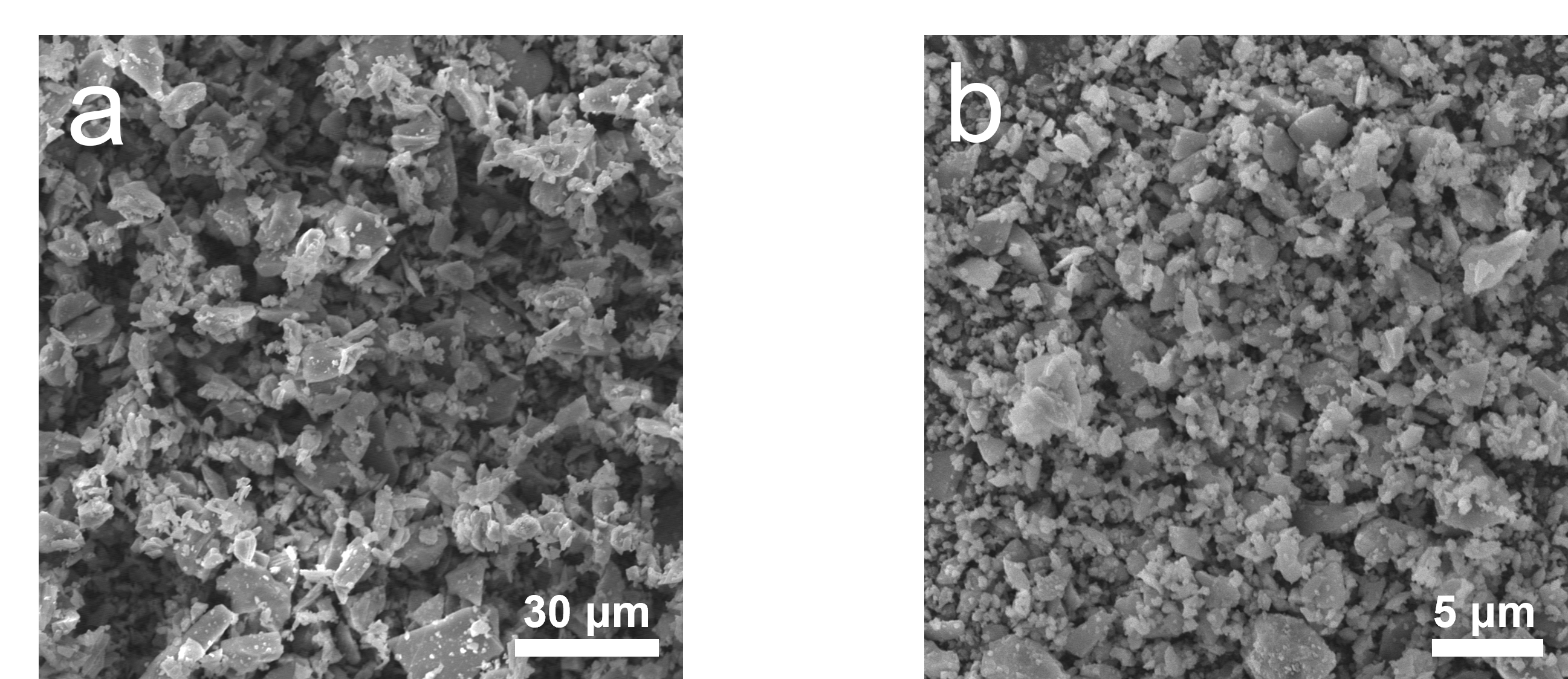


**Fig. S3** SEM images of **commercial Si**

**
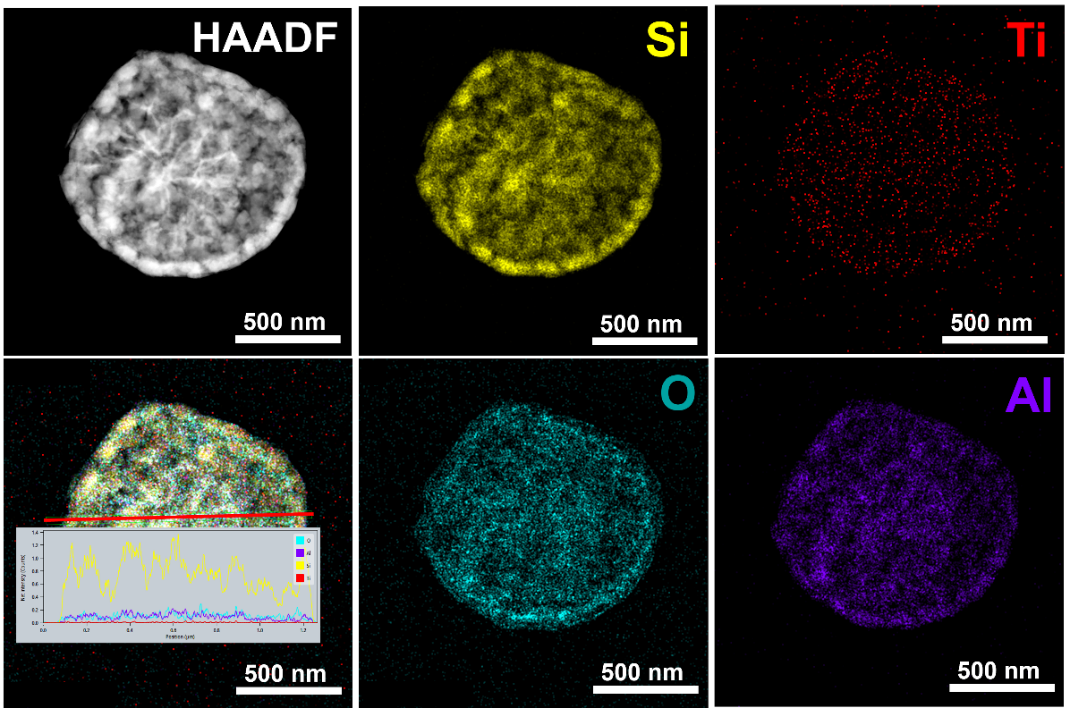
**

**Fig. S4** Elemental mapping images of ***p*-Si@ATO**

**Fig. S5** Nitrogen sorption/desorption isotherms and the corresponding pore size distribution of ***p*-Si**. SSA: 98.5 m^2^ g^−1^


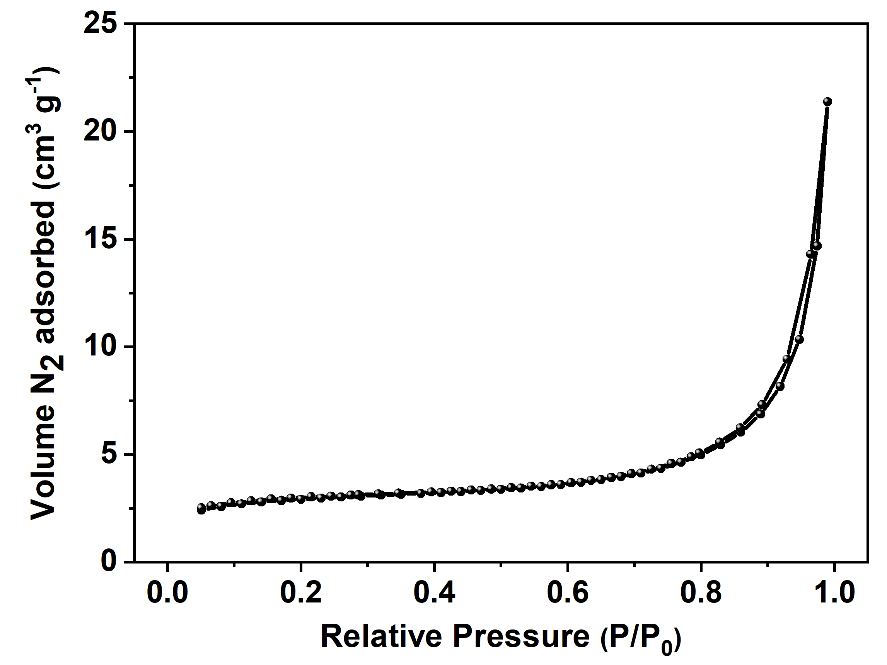


**Fig. S6** Nitrogen sorption/desorption isotherms of **commercial Si**. SSA: 10.1 m^2^ g^−1^


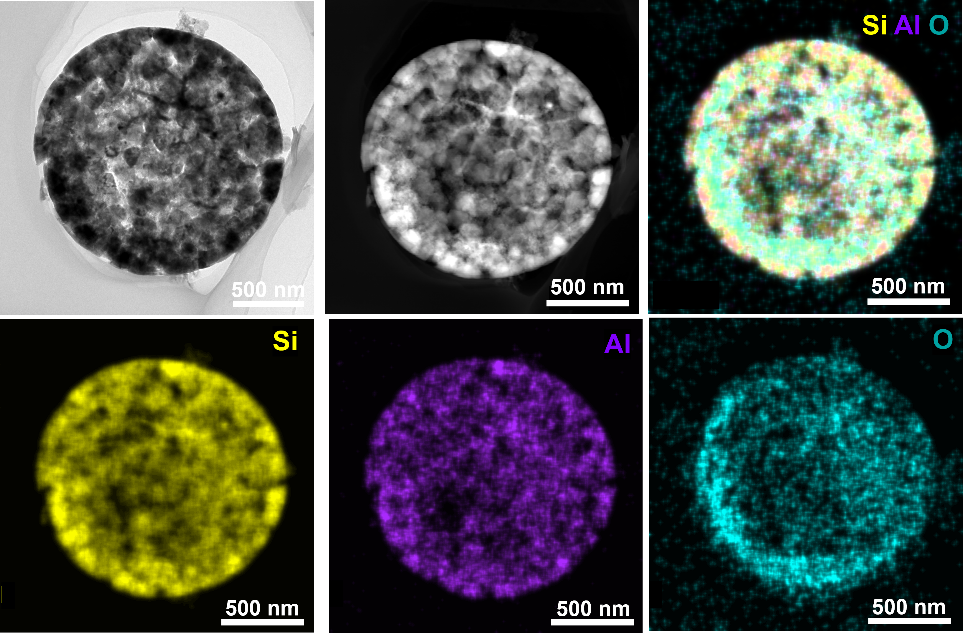


**Fig. S7** TEM and elemental mapping images of ***p*-Si** obtained from acid etching of pristine AlSi_20_ alloy


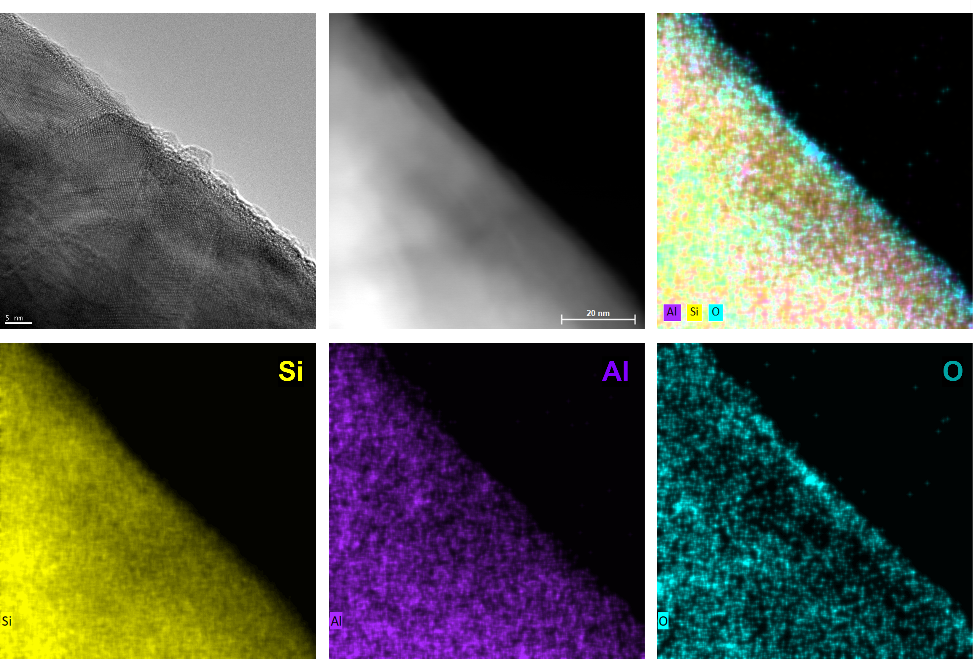


**Fig. S8** HRTEM and elemental mapping images of surface oxide layer on the ***p*-Si**

**
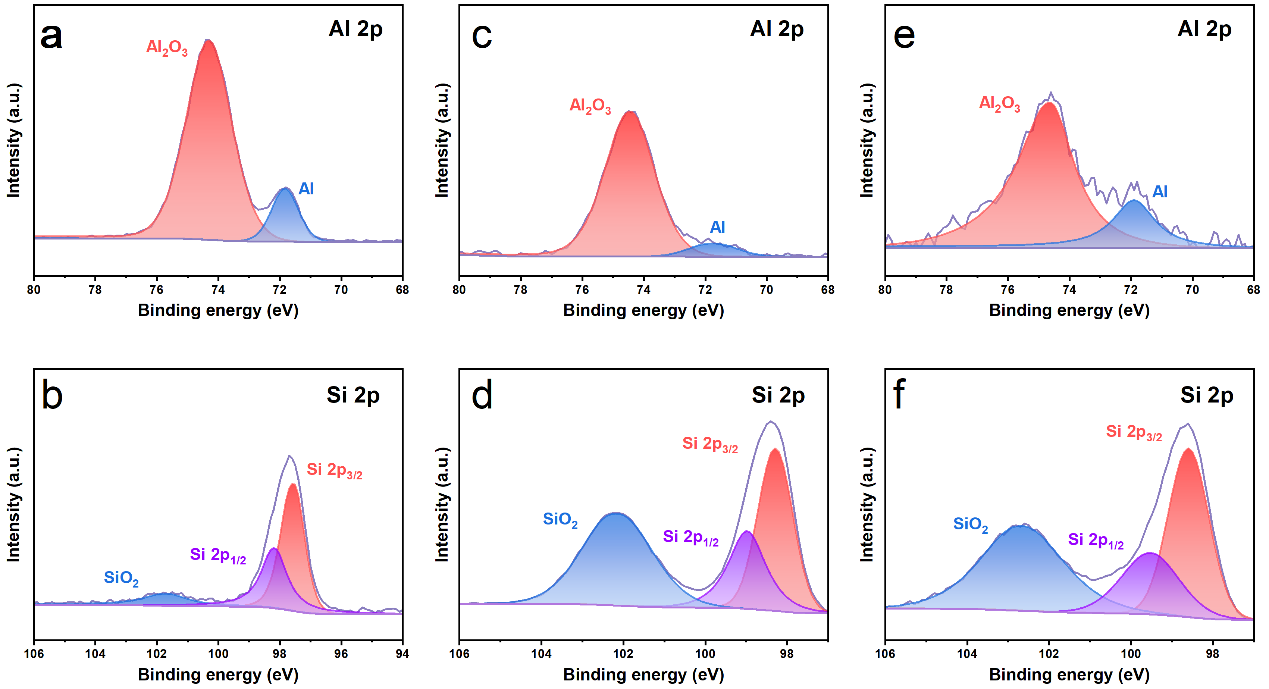
**

**Fig. S9** The Al 2p and Si 2p XPS of AlSi_20_ alloy (**a, b**), *p*-Si@ATO (**c, d**) and *p*-Si (**e, f**)

**
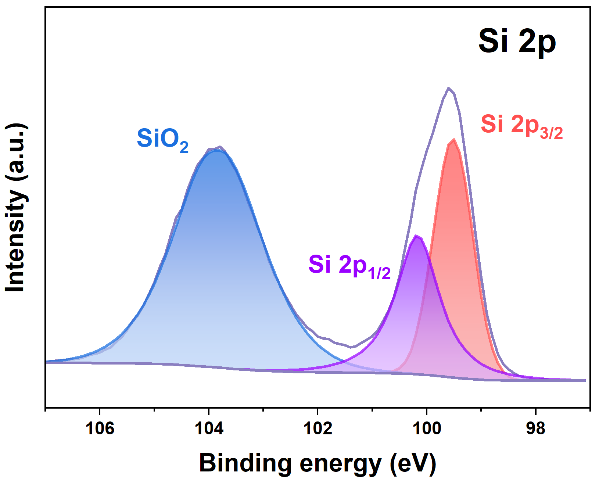
**

**Fig. S10** The Si 2p XPS of **commercial Si**


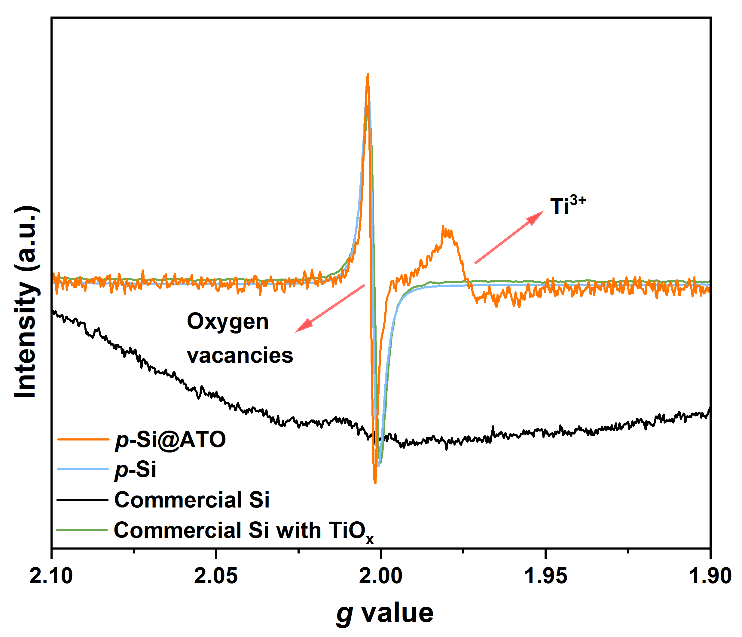
**Fig. S11** EPR spectroscopy of *p*-Si@ATO, *p*-Si, commercial Si and commercial Si with TiO_x_

Commercial Si with TiO_x_: commercial Si replaces AlSi_20_ alloy for hydrolysis of TBOT under the alkaline conditions provided by TMAOH. The results indicate the presence of only oxygen vacancies, and no Ti^3+^ signal was detected in the absence of Al.


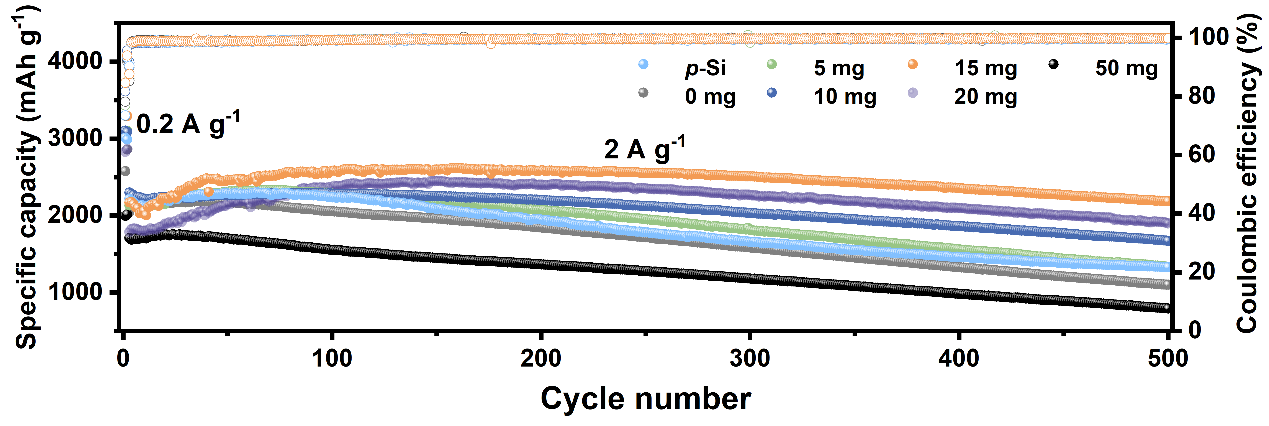
**Fig. S12** Comparison of performance of *p*-Si@ATO with different TBOT mass at 2 A g^−1^

**
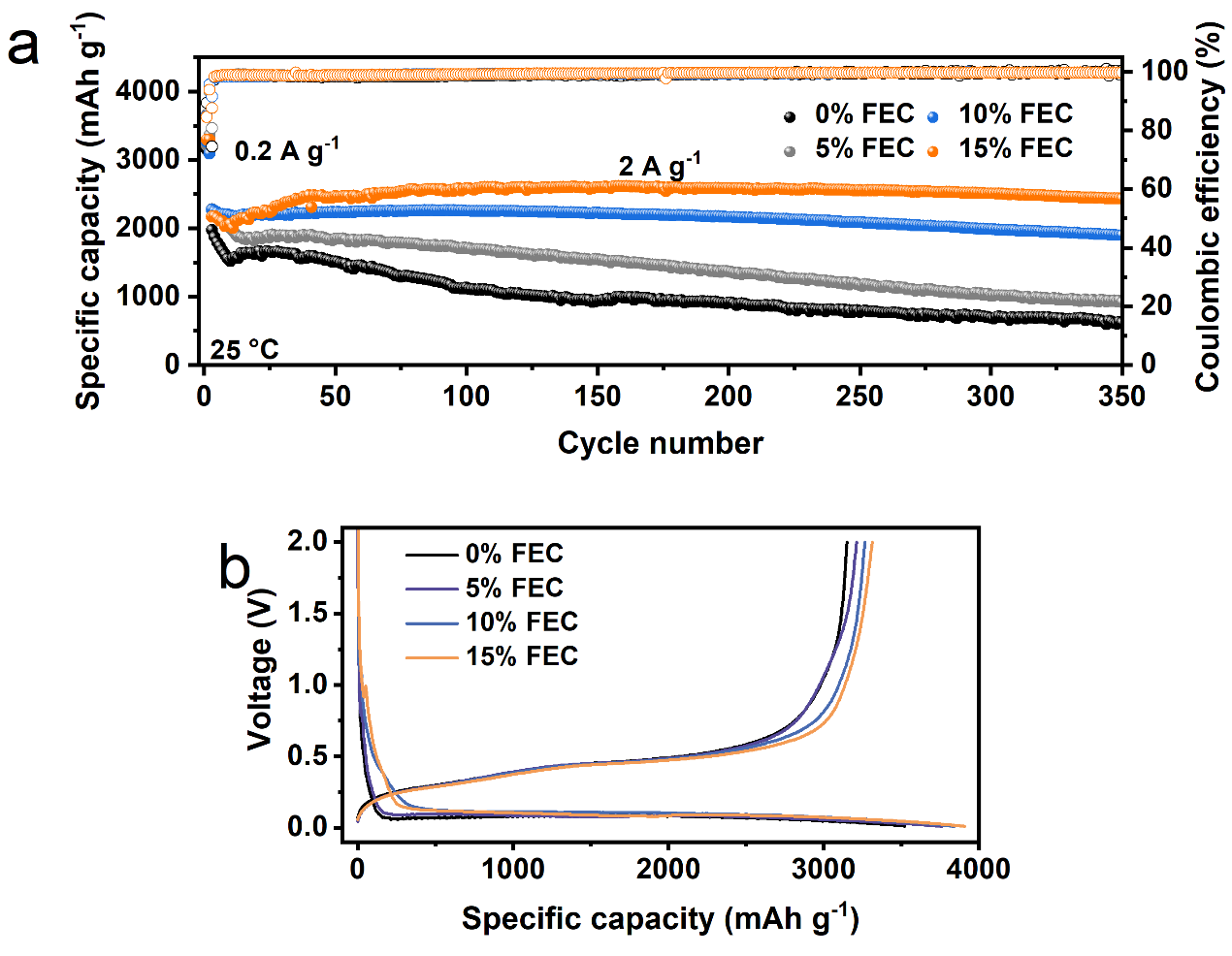
Fig. S13** (**a**) Cycling performance of different FEC concentrations at 2 A g^−1^ after 2 cycles at 0.2 A g^−1^; (**b**) Galvanostatic first cycle charge/discharge voltage profiles at 0.2 A g^−1^


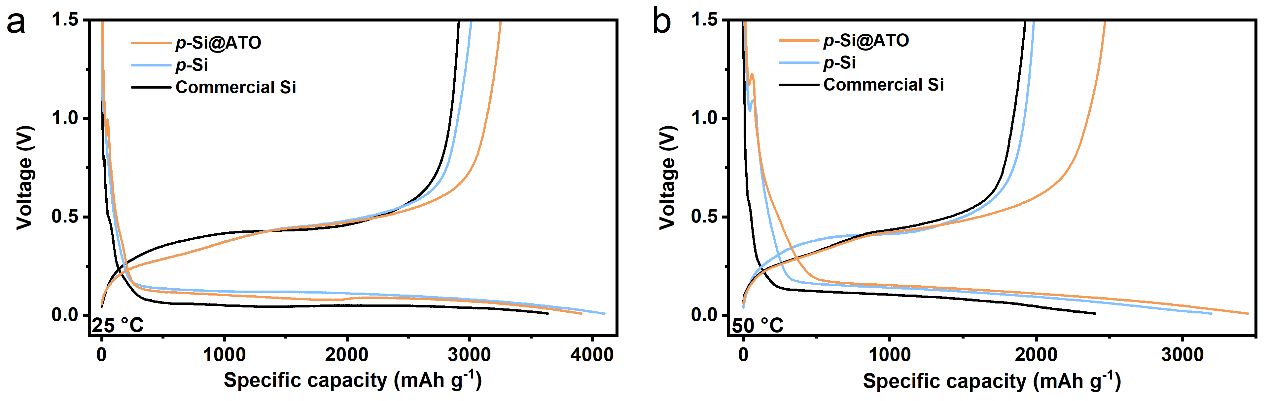
**Fig. S14** Galvanostatic first cycle charge/discharge voltage profiles of *p*-Si@ATO, *p*-Si and commercial Si at 0.2 A g^−1^


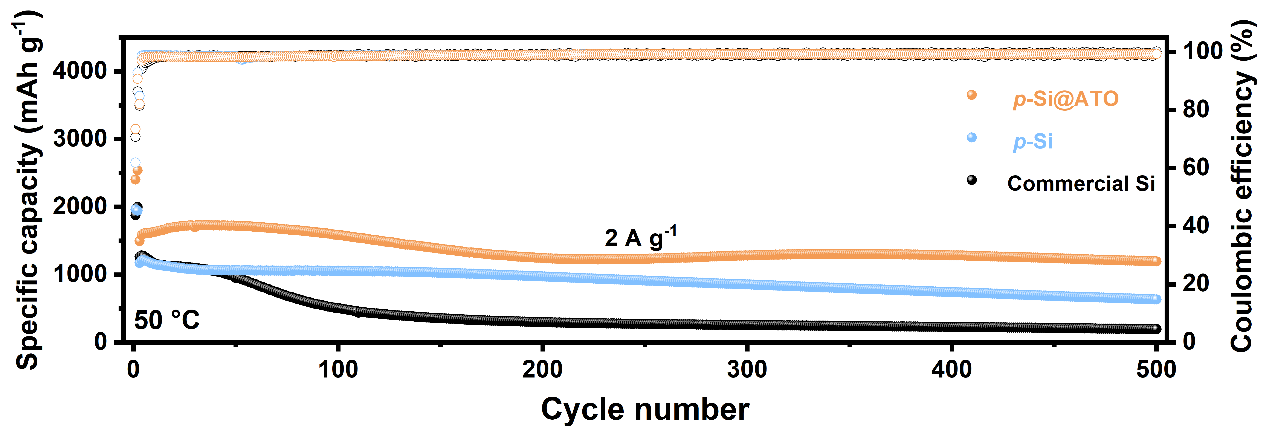


**Fig. S15** Cycling performance of *p*-Si@ATO, *p*-Si and commercial Si at 25 °C

**
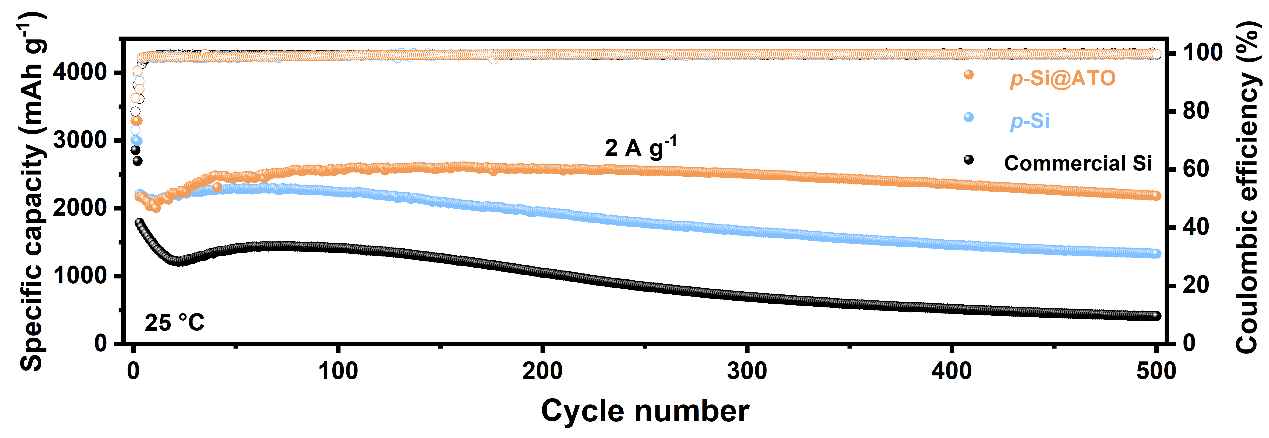
**

**Fig. S16** Cycling performance of *p*-Si@ATO, *p*-Si and commercial Si at 50 °C

**
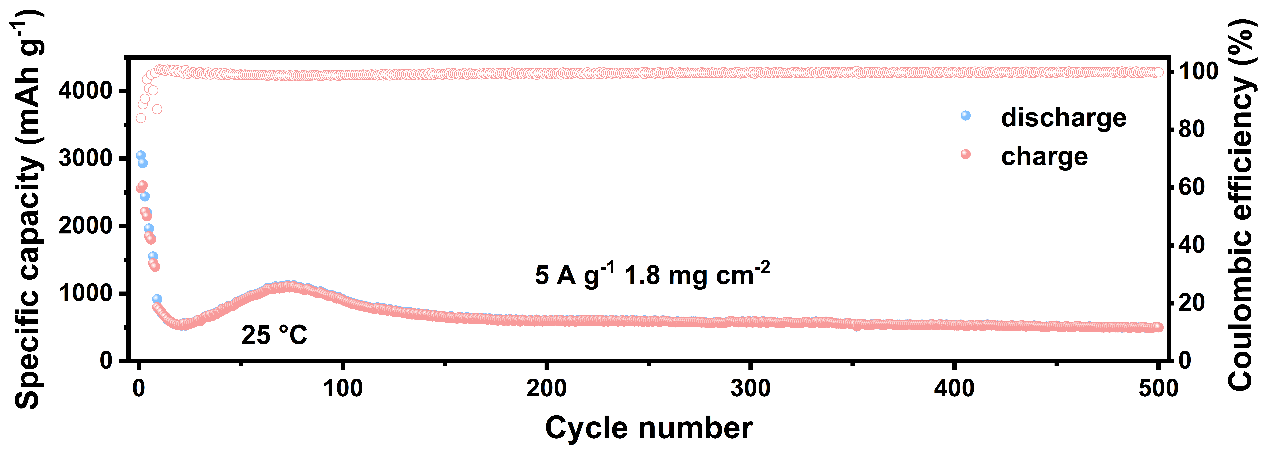
**

**Fig. S17** Cycling performance of *p*-Si@ATO electrode with a load of 1.8 mg cm^−2^ at 25 ℃

The high loading anode of 1.8 mg cm^−2^ (without additional conductive agent is added) displays an area capacity of approximately 3.2 mAh cm^−2^ at 0.2 A g^−1^, and can withstand high current density cycles.


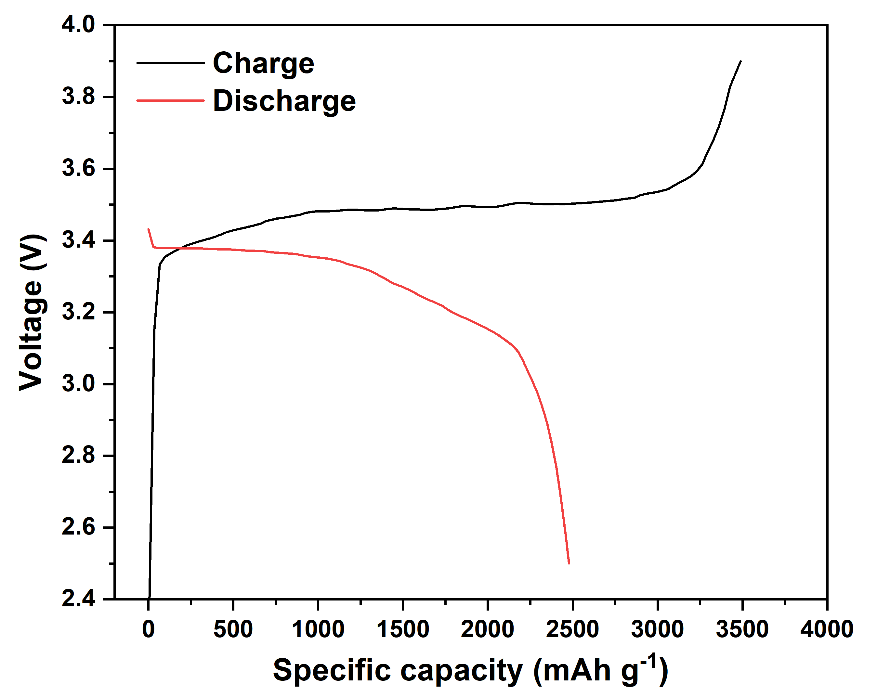


**Fig. S18** Capacity-voltage curves of *p*-Si@ATO||LFP pouch-cell at first cycle

**
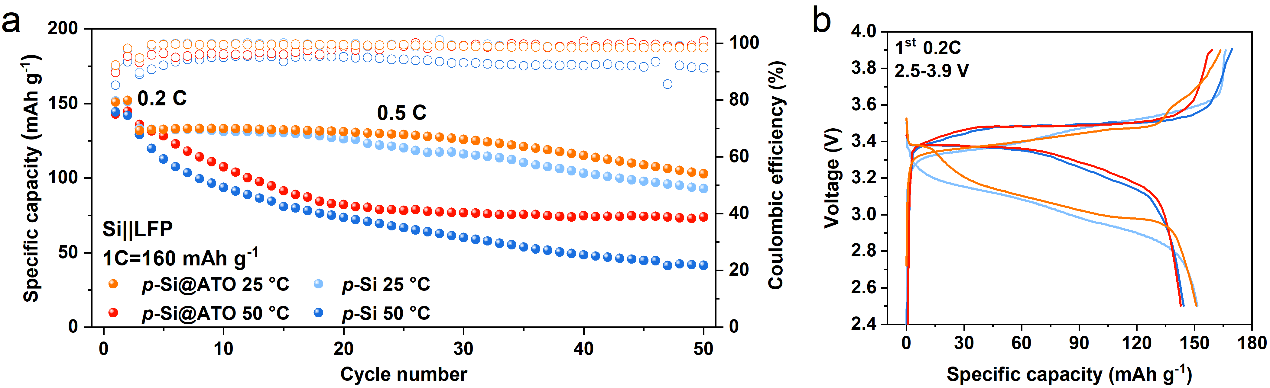
Fig. S19** (**a**) Cycling performance of *p*-Si@ATO||LFP and *p*-Si||LFP coin-type full-cell; (**b**) Capacity-voltage curves of first cycle at 0.2 C

The coin-type full-cell has an N/P ratio of approximately 1.05, with a voltage range of 2.5–3.9 V and 1 C =160 mAh g^−1^. The negative electrode is pre-cycled 5 times at 0.2 A g^−1^ before being assembled into the full-cell.


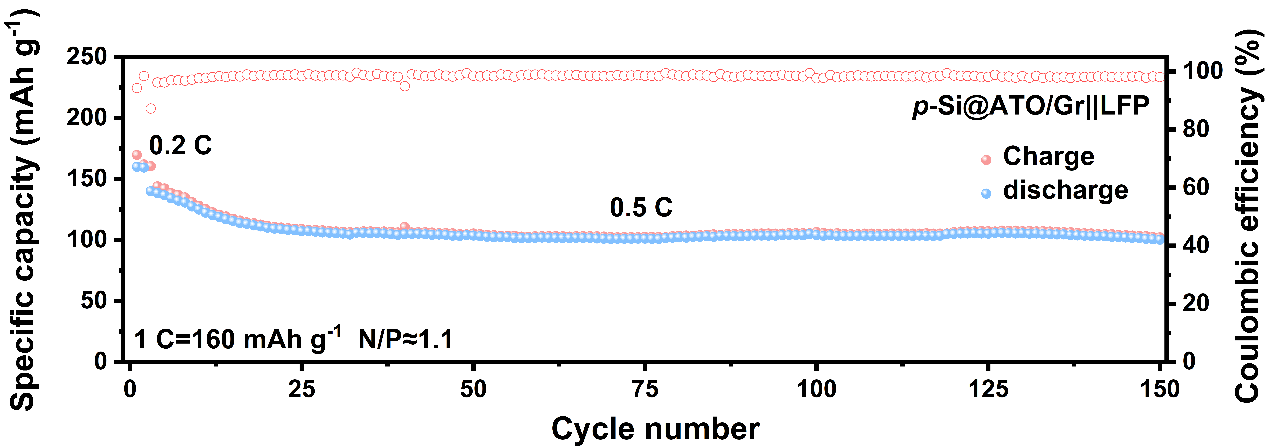


**Fig. S20** Cycling performance of coin-type full-cell *p*-Si@ATO/Gr||LFP at 25 ℃

The *p*-Si@ATO/Gr electrode slurry was fabricated by mixing 76.5 wt% graphite, 8.5 wt% *p*-Si@ATO powder, 10 wt% polyacrylic acid, and 5 wt% acetylene black in N-methyl-2-pyrrolidone. The initial Coulombic efficiency is 94.3% at 0.2 C.

**
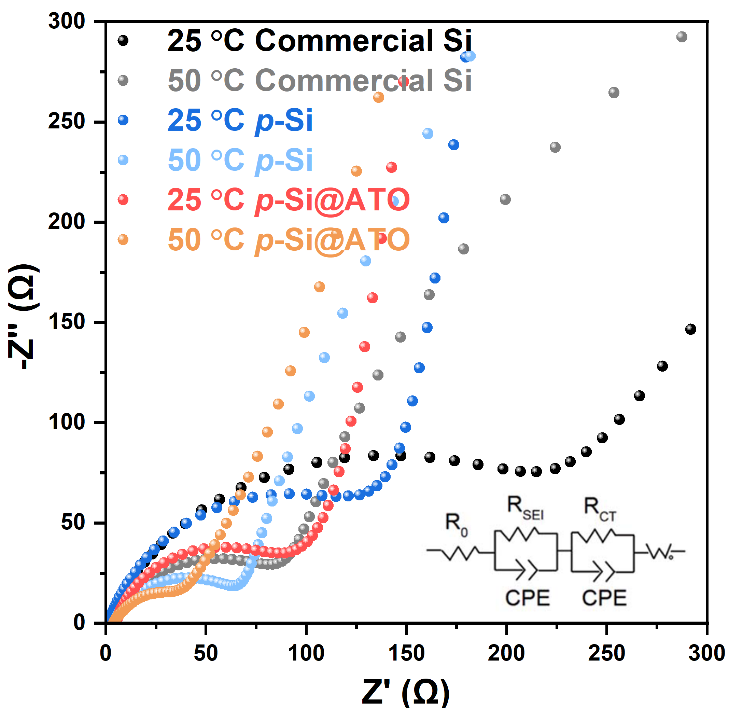
**

**Fig. S21** EIS after 1 cycle measured at 100% SOC

**
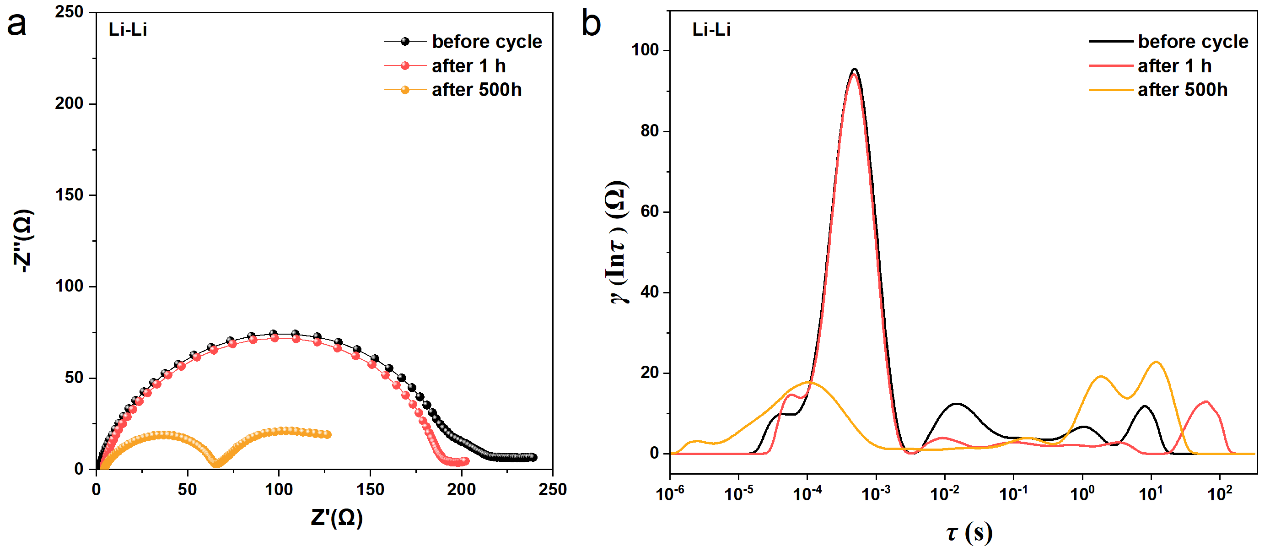
Fig. S22** (**a**) EIS of Li-Li symmetric cells and (**b**) the corresponding DRT


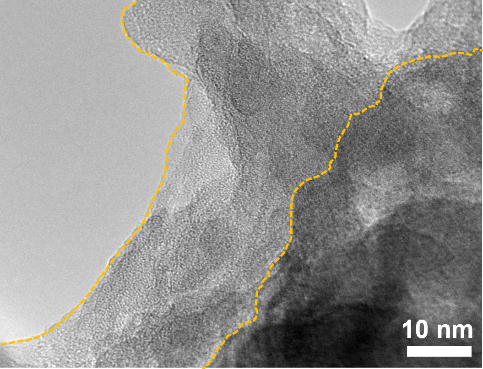


**Fig. S23** HRTEM images of *p*-Si electrodes after 100 cycles


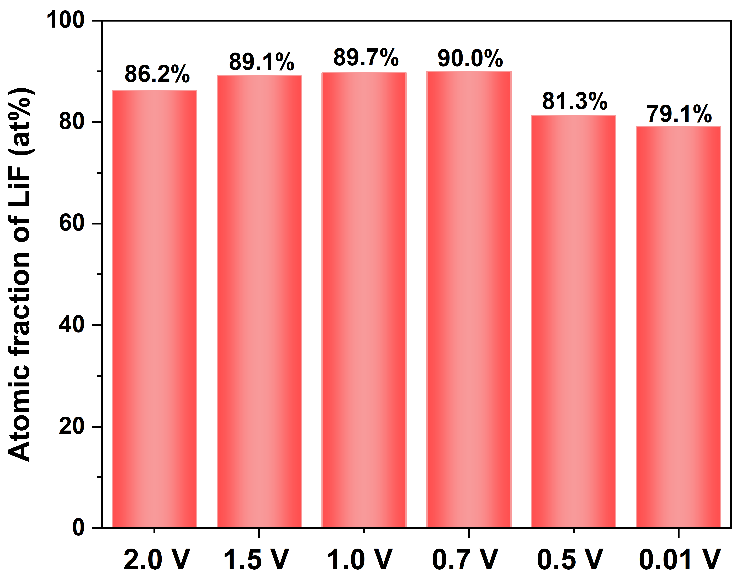


**Fig. S24** F at% of LiF in F 1s XPS at different voltages


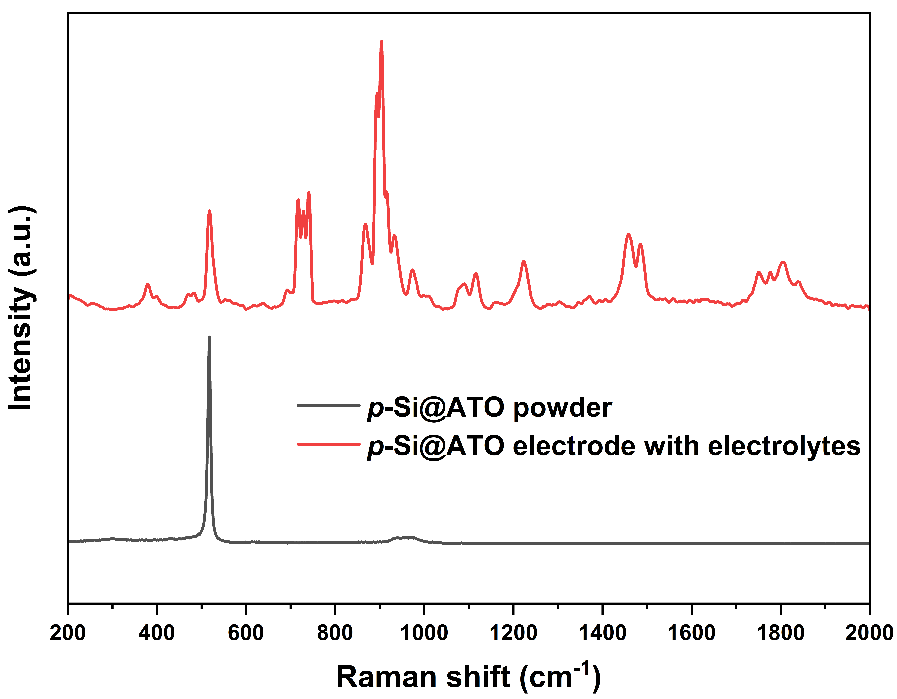


**Fig. S25** Raman spectroscopy of *p*-Si@ATO powder and *p*-Si@ATO electrode before cycle


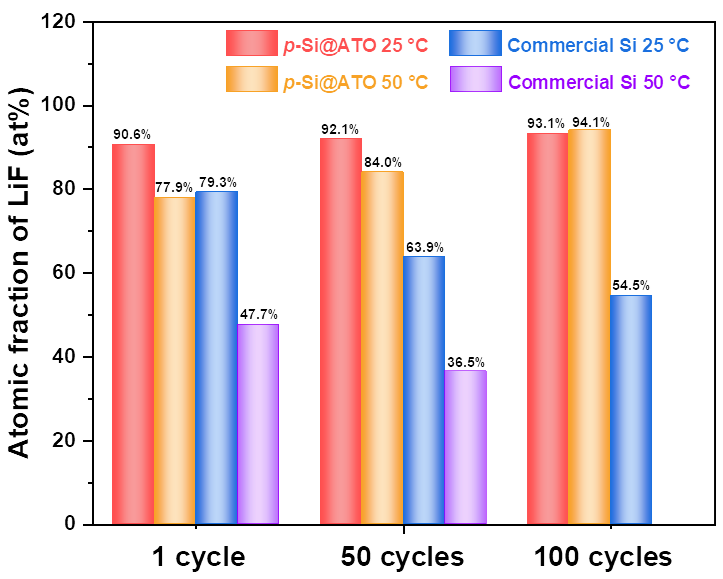


**Fig. S26** F at% of LiF in F 1s XPS after 1, 50 and 100 cycles


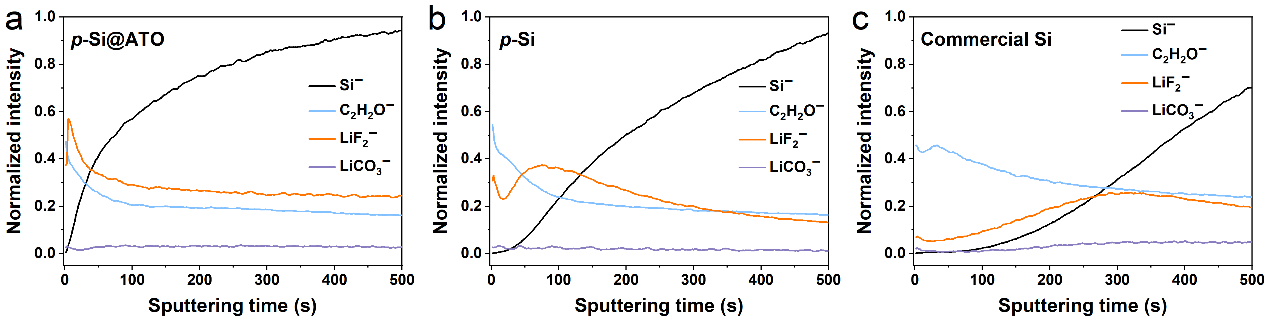
**Fig. S27** ToF-SIMS results of the Si electrode after 100 cycles: depth profiles of various species

The ions composition and distribution of the anode surface and depth sputtering area (70×70 μm^2^) were characterized by Time-of-Flight secondary ion mass spectrometry (ToF-SIMS, ToF-SIMS 5 ION-ToF GmbH, Germany). ToF-SIMS was equipped with a 30 keV Bi^3+^ primary ion gun and a 1 keV Cs^+^ sputter gun, and an electron flood gun was used for charge neutralization. The silicon-based electrodes were tested after 100 cycles and subsequently washed repeatedly with DMC.


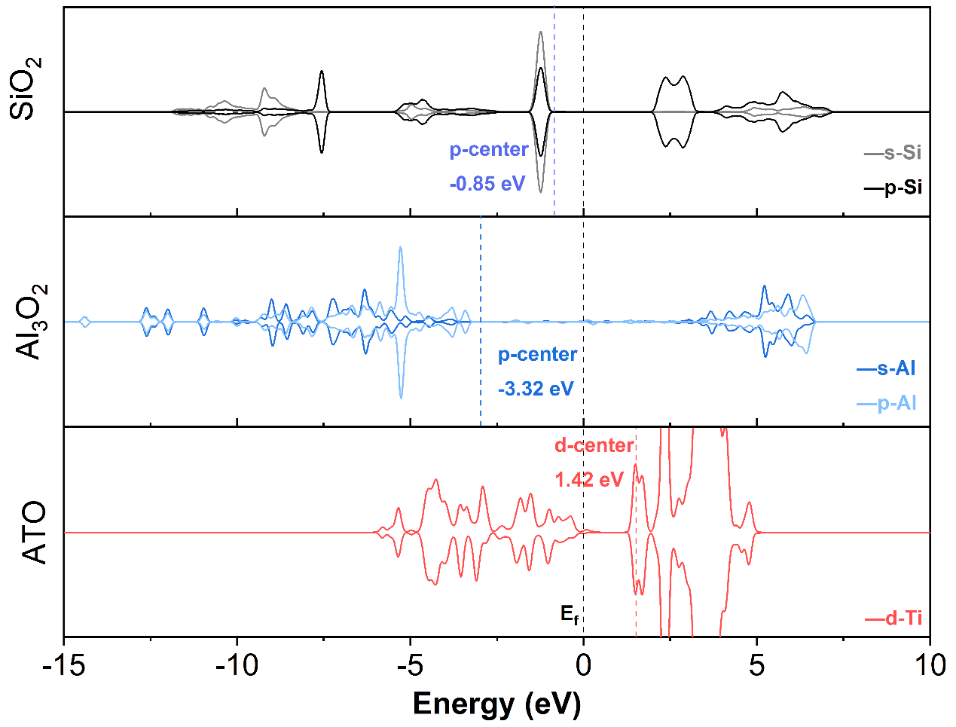


**Fig. S28** PDOS without FEC on SiO_2_, Al_2_O_3_ and ATO surfaces

**Table S1** Content of elements in ***p*-Si@ATO**

| **Element** | **EDS Mapping (wt%)** | **ICP (wt%)** |
| --- | --- | --- |
| **Si** | 46.2 | 85.11 |
| **Al** | 35.3 | 5.58 |
| **Ti** | 0.14 | 0.32 |

**Table S2** Content of elements after the etching process with alkaline solution

| **Element** | **After TMAOH etching (wt%)** |
| --- | --- |
| **Si** | 46.16 |
| **Al** | 35.28 |
| **Ti** | 0.14 |

**Table S3** Comparison of the electrochemical characteristics for Si-base anode materials at high temperture

| **Anode material** | **Electrolytes** | **Specific capacity (temperature)** | **Capacity retention** | **References** |
| --- | --- | --- | --- | --- |
| ***p*-Si@ATO** | 1 M LiPF_6_ in EC/DEC (1:1) + 15% FEC | 2423 mAh g^−1^ at 0.2 A g^−1^,  270 mAh g^−1^ at 25 A g^−1^ (50 ℃) | 80.0% after 500 cycles at 2 A g^−1^ | **This work** |
| Microsized Si | 2 M LiFSI in Pyr_14_FSI | ~3500 mAh g^−1^ at 0.4 A g^−1^ (80 ℃) | 85%  after 100 cycles at 4 A g^−1^ | [S1] |
| Si@GCA13 | 1 M LiPF_6_ in EC/DEC  (1:1) + 10% FEC and 1%VC | 2302 mAh g^−1^ at 0.8 A g^−1^ after 55 cycles (60 ℃) | \ | [S2] |
| MFG/PNSi@C | 1 M LiPF_6_ in EC/DC/DMC (1:1:1) | 851 mAh g^−1^ at 0.4 A g^−1^ (50 ℃) | \ | [S3] |
| Si@C@Al_2_O_3_ | 1 M LiPF_6_ in EC/EMC  (3:7) + 5% FEC | 1276 mAh g^−1^ at 8 A g^−1^ (80 ℃) | \ | [S4] |
| 3D-Si@SiOx/C | 1 M LiPF_6_ in EC/EMC  (1:2) + 2% FEC | 1746 mAh g^−1^ at 0.2 A g^−1^ (60 ℃) | 79.4% after 70 cycles at 0.2 A g^−1^ | [S5] |
| Si@BaTiO_x_-600@C | 1.3 M LiPF_6_ in EC/DEC  (3:7) + 10% FEC | 2021 mAh g^−1^ at 1 C (60 °C) | 64% after 600 cycles at 1 C | [S6] |
| Si-C@Al_2_O_3_ | 1 M LiPF_6_ in EC/DMC  (1:1) + 10% FEC | 668.6 mAh g^−1^ at 0.5 A g^−1^ (60 °C) | 80.4% after 100 cycles at 0.5 A g^−1^ | [S7] |
| Si@PAA-B-HPR | 1 M LiPF_6_  in EC/DEC (1:1) + 5% FEC and 0.5% VC | 2106 mAh g^−1^ at 1.4 A g^−1^ (55 °C) | 50.14% after 500 cycles at 1.4 A g^−1^ | [S8] |
| Si@C-5CNTs | 1 M LiPF_6_ in EC/DMC (1:1) | 819 mAh g^−1^ at 2 A g^−1^ (40 °C) | 83.6% after 100 cycles at 0.1 A g^−1^ | [S9] |

**Supplementary References**

1. Q. Liu, T. Meng, L. Yu, S. Guo, Y. Hu et al., Interface engineering to boost thermal safety of microsized silicon anodes in lithium-ion batteries. Small Methods. **6**, 2200380 (2022). https://doi.org/10.1002/smtd.202200380
2. Y. Tong, S. Jin, H. Xu, J. Li, Z. Kong et al., An energy dissipative binder for self-tuning silicon anodes in lithium-ion batteries. Adv Sci **10**, 2205443 (2023). https://doi.org/10.1002/advs.202205443
3. H. Chen, X. Hou, F. Chen, S. Wang, B. Wu et al., Milled flake graphite/plasma nano-silicon@carbon composite with void sandwich structure for high performance as lithium ion battery anode at high temperature. Carbon. **130**, 433-440 (2018). https://doi.org/10.1016/j.carbon.2018.01.021
4. L. Fu, A. Xu, Y. Song, J. Ju, H. Sun et al., Pinecone-like silicon@carbon microspheres covered by Al_2_O_3_ nano-petals for lithium-ion battery anode under high temperature. Electrochim Acta. **387**, 138461 (2021). https://doi.org/10.1016/j.electacta.2021.138461
5. J. Lee, J. Moon, S. A. Han, J. Kim, V. Malgras et al., Everlasting living and breathing gyroid 3D network in Si@SiO_x_/C nanoarchitecture for lithium ion battery. ACS Nano. **13**, 9607-9619 (2019). https://doi.org/10.1021/acsnano.9b04725
6. H. Park, S. Choi, S.-J. Lee, Y.-G. Cho, G. Hwang et al., Design of an ultra-durable silicon-based battery anode material with exceptional high-temperature cycling stability. Nano Energy. **26**, 192-199 (2016). https://doi.org/10.1016/j.nanoen.2016.05.030
7. C. Zhang, F. Ji, D. Li, T. Bai, H. Zhang et al., Interface engineering enables wide-temperature Li-ion storage in commercial silicon-based anodes. Small. 2310633 (2024). https://doi.org/10.1002/smll.202310633
8. Z. H. Xie, M. Z. Rong, M. Q. Zhang, Dynamically cross-linked polymeric binder-made durable silicon anode of a wide operating temperature Li-ion battery. ACS Appl Mater Interfaces. **13**, 28737-28748 (2021). https://doi.org/10.1021/acsami.1c01472
9. M. Zhang, L. Zhao, D. Sun, Y. Sun, C. Xu et al., S doped CNTs scaffolded Si@C spheres anode toward splendid high-temperature performance in lithium-ion battery. Appl Surf Sci. **626**, 157254 (2023). https://doi.org/10.1016/j.apsusc.2023.157254
